# The Interfacial Interactions of Glycine and Short Glycine Peptides in Model Membrane Systems

**DOI:** 10.3390/ijms22010162

**Published:** 2020-12-26

**Authors:** Kaitlin A. Doucette, Prangthong Chaiyasit, Donn L. Calkins, Kayli N. Martinez, Cameron Van Cleave, Callan A. Knebel, Anan Tongraar, Debbie C. Crans

**Affiliations:** 1 Cell and Molecular Biology Program, Colorado State University, Fort Collins, CO 80523, USA; Kaitlin.Doucette@colostate.edu; 2 Department of Chemistry, Colorado State University, Fort Collins, CO 80523, USA; dlcalkinscsu@gmail.com (D.L.C.); kmarti11@berkeley.edu (K.N.M.); Cameron.Van_Cleave@colostate.edu (C.V.C.); callan.knebel@rams.colostate.edu (C.A.K.); 3 School of Chemistry, Institute of Science, Suranaree University of Technology, Nakhon Ratchasima 30000, Thailand; aomchaiyasit@gmail.com (P.C.); anan_tongraar@yahoo.com (A.T.)

**Keywords:** glycine, reverse micelles, AMPs, pK_a_, ^1^H NMR

## Abstract

The interactions of amino acids and peptides at model membrane interfaces have considerable implications for biological functions, with the ability to act as chemical messengers, hormones, neurotransmitters, and even as antibiotics and anticancer agents. In this study, glycine and the short glycine peptides diglycine, triglycine, and tetraglycine are studied with regards to their interactions at the model membrane interface of Aerosol-OT (AOT) reverse micelles via ^1^H NMR spectroscopy, dynamic light scattering (DLS), and Langmuir trough measurements. It was found that with the exception of monomeric glycine, the peptides prefer to associate between the interface and bulk water pool of the reverse micelle. Monomeric glycine, however, resides with the N-terminus in the ordered interstitial water (stern layer) and the C-terminus located in the bulk water pool of the reverse micelle.

## 1. Introduction

Small peptides play an essential role in a variety of biological functions, acting as chemical messengers, intra- and intercellular mediators, hormones, and neurotransmitters [1,2,3]. Peptides also play an important role as antibiotics, such as bacitracin and colistin, as well as antimicrobial peptides (AMPs, also referred to as host defense peptides) [4,5,6]. AMPs are peptides produced by multicellular organisms as part of the innate immune response found in all classes of life and function as a defense against pathogenic microbes. They exert this function in a number of ways, such as the suppression of biofilm formation, induction of the dissolution of existing biofilms, and attracting phagocytes via chemotaxis to induce non-opsonic phagocytosis [5,7,8]. In addition to their antimicrobial function, recently, it has been found that AMPs may also have anticancer activity; they are able to trigger cytotoxicity of a number of cancer cells through the interaction of the amphipathic or cationic peptide with the plasma membrane of the cell, which selectively exposes negatively charged phosphatidylserine lipids [9,10]. The combination of the function of AMPs as antimicrobial agents as well as anticancer agents makes them a promising starting point for antimicrobial and anticancer drug design [11,12,13,14].

**Figure 1:**
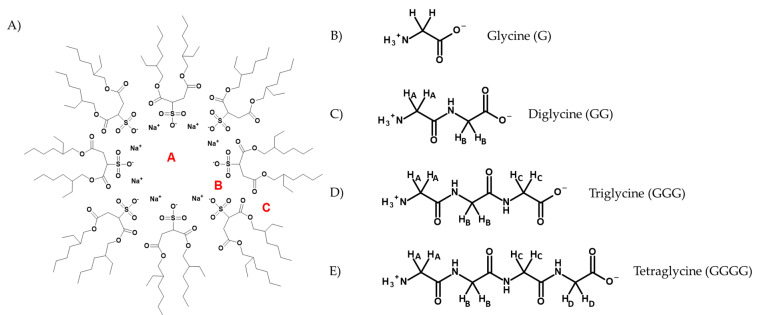
The structure of glycine (G) analogs and schematic of the Aerosol-OT reverse micelle (AOT RM) model system. (**A**) A schematic of a simplified structure of an RM. “A” represents the bulk water pool, “B” is the interfacial region of the RM in the region of the charged AOT head groups, and “C” represents association in the more hydrophobic region of the RM in the region of the acyl groups, hydrophobic tails, and isooctane solvent. (**B**) The amino acid G at physiological pH. (**C**) Structure of diglycine (GG), with protons labeled corresponding to proton peaks analyzed by ^1^H NMR (**D**) Structure of triglycine (GGG) with protons labeled corresponding to proton peaks analyzed via ^1^H NMR. (**E**) Structure of tetraglycine (GGGG), with protons labeled corresponding to proton peaks analyzed via ^1^H NMR.

In order to exert their antimicrobial or anticancer properties, AMPs must interact with the plasma membrane of the bacterial or cancer cell [5,15]. This interaction with the membrane is associated with their mechanism of action, which can include disruption of the membrane, disruption of membrane-associated physiological processes such as cell wall synthesis, or even translocation across the membrane for interaction with a cytoplasmic target [5,16,17,18]. The interactions of these small peptides are dependent on a variety of variables such as size, amino acid composition, secondary structure, and amphiphilic behavior, and their mechanism of action is generally unknown with the exceptions of a few representative examples [8,9,19,20]. Additionally, AMP interactions with the membrane depend on the composition of the membrane itself, as they tend to be attracted more to negatively charged membranes such as bacterial membranes or plasma membranes of cancer cells, which selectively expose negatively charged phosphatidylserine lipids [10,21]. Because of this, AMPs prefer membranes with a high concentration of anionic lipids, those that maintain a high electrical potential gradient, and membranes that tend to lack cholesterol [5,22,23]. It is thus important to study the interactions of peptides at a membrane interface using a small representative amino acid and a membrane mimetic interface (Figure 1A) to determine the molecular placement of the molecules at the membrane as well as the manner by which they interact.

Of the twenty amino acids that are found in peptides, glycine (G, Figure 1B) is both the smallest and the most versatile [24]. Having only a hydrogen atom as its substituent, it is the only amino acid that is achiral, and as such, it is compatible with hydrophilic environments, and although it is not directly soluble in for example isooctane (Figure S1) it can partition toward hydrophobic regions in inhomogenous environments. In addition, it has many biological functions, one of the most notable of which as a simple inhibitory and excitatory neurotransmitter, and as such, it is a logical representative amino acid for investigation of simple peptide and amino acid interactions with a membrane, and in addition, there have been numerous reports of glycine-rich AMPs [25,26,27,28].

Because we are interested in obtaining molecular information on how simple peptides interact with membrane interfaces, we will use monomeric, dimeric, trimeric, and tetrameric G-containing peptides, hereafter referred to as G, GG, GGG, and GGGG (Figure 1B–E). To study how these small peptides behave near cellular membranes, we use a reverse micellar (RM) system (Figure 1A) which consists of a self-assembled ternary system containing surfactant, organic solvent, and water [29,30,31,32]. The surfactant, in this case, is Aerosol-OT (AOT), also known as sodium 2-diethylhexylsulfosuccinate, which arranges itself such that the water pool is contained by the negatively charged head groups of the AOT, and surfactant tails extend outward into the organic solvent—in this case, isooctane (2,2,4-trimethylpentane) [33,34]—and commonly, water droplets contained in this system range from a size of 1 to 10 nm [35,36]. The RM system provides both a hydrophilic and hydrophobic environment at a negatively charged interface, making it a good model system to investigate the interactions of molecules with membrane interfaces [37,38].

To investigate the interactions of G and G-containing peptides with model membranes, it is of interest to determine its location within the RM. That is, whether it is located near the charged AOT heads, bulk water pool, or in the ordered, interstitial water between the charged interface and the water pool, referred to as the stern layer [39]. Furthermore, the location of the molecule of interest may be sensitive to the local pH of the RM interior [40,41].

In this study, we use AOT RMs and Langmuir monolayers to gain insight into how G and G-based peptides interact with simple membrane model systems. Specifically, we investigate here the interaction of G, GG, GGG, and GGGG with an AOT RM interface and with dipalmitoyl phosphatidylcholine (DPPC) and dipalmitoyl ethanolamine (DPPE) monolayers to determine the interactions and placement of G compounds at a model membrane interface to mimic non-cancerous and human cells.

## 2. Results

The chemical shifts of G, GG, GGG, and GGGG were examined by ^1^H NMR spectroscopy to compare their chemical shifts in aqueous solution with those peaks obtained in the environment of the RM model membrane (Section 2.1, Section 2.2, Section 2.3 and Section 2.4). Solutions containing each of the G compounds were made at varying pH values to determine the pK_a_ values of each in both aqueous environment and in the environment of the RM (summarized in the Discussion section, Figure S2), and representative NMR spectra for each compound in RM and D_2_O as well as exact chemical shift values are given in the Supplemental Materials. Each of these compounds showed a slight difference in chemical shift values between RMs and the compound alone in D_2_O, indicating a difference in environment for the probe molecule. These data give some structural information about the location of the probe within the reverse micelle. The systems were also investigated using dynamic light scattering (DLS) to verify formation of the RMs and to examine the impact of the G compounds on the RM system (Section 2.5).

In Section 2.6, we further support the observations made in this paper in Section 2.1, Section 2.2, Section 2.3, Section 2.4 and Section 2.5 by using Langmuir trough measurements. These studies used a natural lipid as well as a different method, and this was done to investigate whether the conclusion obtained by using the microemulsions system could be confirmed and extended to studies of physiological lipids and human cells.

### 2.1. ^1^H NMR Spectroscopy of L-Glycine (G) in RM

**Figure 2:**
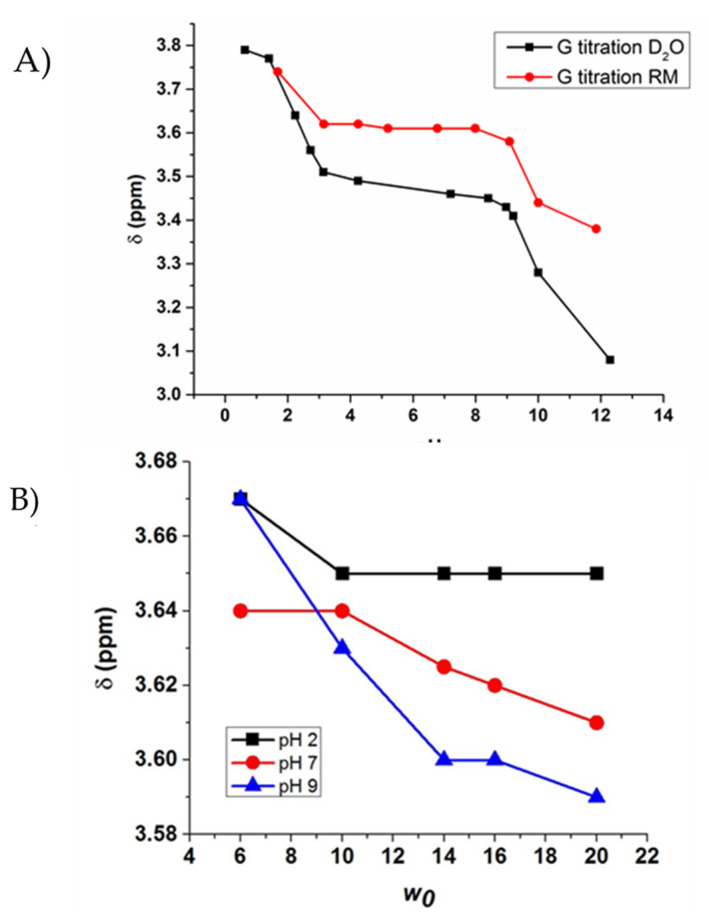
(**A**) Comparison of ^1^H NMR chemical shifts of G in aqueous and RM environments. (**B**) The ^1^H NMR chemical shift of G is plotted with increasing *w_0_* at pH 2, 7, and 9. Error bars have been included in the plot; however, due to minimal error, they are not visible beyond the symbols used in the graph.

A series of samples with RMs of size *w_0_* 10 (where *w_0_* = [AOT]/[H_2_O]) were made containing G at varying pH values by adding 200 mM G solution in D_2_O at the pH specified to the appropriate volume of 750 mM AOT solution dissolved in isooctane. The chemical shifts of these were recorded using ^1^H NMR spectroscopy and chemical shifts are compared with the representative spectra shown (Figure 2A, Figures S2–S6). The pK_a_ values were calculated (Figure S2) from the spectra both in D_2_O and in microemulsions are listed in Table 2 in the Discussion section. Values obtained from G in aqueous and RM environments show that the pK_a_ of the C-terminus differs very little between aqueous and RM environments, but the N-terminus differs significantly, with a pK_a_ value of 10.7 in D_2_O and 8.51 in the RM model membrane. This difference, or lack thereof, in pK_a_ values between the two environments gives some information about the environments surrounding the carboxy- and amine-terminal ends of G within the RM [42]. Because there is little change between the carboxyl pK_a_ in RM and D_2_O, this suggests that this portion of the compound is in an environment that is the same. In the context of the RM model system, this observation is consistent with the C-terminus being in the stern layer/aqueous environment directed toward the bulk water pool (Figure 1A). The significant decrease in pK_a_ between aqueous and RM environments for the amine-terminal end of G indicates a significant change in environment, such that the amine-terminal end is located near or in the charged region of the RM interface.

It is likely that the experimental N-terminal pK_a_ of G in RM is lower than what is reported in the literature due to the higher ionic strength near the charged interface. In pure aqueous solution, the amine is free to hydrogen-bond to the carboxyl moiety of the amino acid, forming an energetically favorable five-membered ring and stabilize the amine. However, in high ionic strength solutions, this H-bonding may be disrupted by the presence of counterions, which are known to accumulate near the interface of the AOT (Figure 1A) [43,44], lowering the pK_a_ of the N-terminus. Additionally, this H-bonding phenomenon could be disrupted by the interaction of the amine with the sulfonate groups on the AOT head groups. This disruption of H-bonding is consistent with the lowering of the pK_a_ values in the reverse micelle, which contains more Na^+^ ions, the presence of charged sulfonate groups, and, therefore, a higher ionic strength.

To further support this conclusion, experiments were performed in which the size of the RM containing the G solutions was varied so that the chemical shifts could be analyzed as a function of increasing vesicle size to give further information about their placement within the RM system. The pH of the G solutions used to prepare the RMs was varied at representative pH values: alkaline pH (pH 9), neutral/physiological pH (pH 7.4), and acidic pH (pH 2). From this experiment, it was found that as the size of the water pool in the RM increased, the chemical shift of the G peak in the neutral- and alkaline-pH RM environments decreased and approached its shift value in D_2_O alone, which is 3.55 at pH 2 and 7.4 (Figure 2B). This suggests that the neutral and negatively charged forms of G, predictably, are not attracted to the interfacial region of the RM due to their charges not interacting with the negatively charged AOT heads [45]. As the vesicle size increases, the interstitial water region becomes less ordered and more analogous to bulk water, and as a result, the compounds that are not highly attracted to the polar interface begin to transition to water that behaves more as bulk water. However, in the case of G at pH 2, the carboxylate moiety is fully protonated, leading to an overall +1 charge of the molecule. As a result, the positive charge of the molecule interacts with the negatively charged polar heads of the AOT consistently, leading to the plateau in chemical shift as the size of the RM increases.

To test the hypothesis that as the vesicle size increases at neutral and alkaline pH, the chemical shift of G approaches that of its shift in pure aqueous environment, experiments were performed in which the pK_a_ of G was calculated in a *w_0_* 30 RM (12.4-nm diameter) instead of the *w_0_* 10 (6.8-nm diameter) that was previously used [35]. These experiments showed that the carboxy-terminal pK_a_ in this larger vesicle stayed the same at 2.5, but the amine-terminal pK_a_ decreased significantly to 9.6 from 8.51, a value much closer to the pK_a_ when G is in an aqueous environment under ionic strength (Figure 2). This is consistent with our hypothesis that G is likely positioned such that the N-terminus is in the interstitial water region of the RM facing the negatively charged interface, while the C-terminus is located closer to the bulk water pool of the RM [45]. As the size of the RM increases, the interstitial water region becomes less ordered and behaves more as bulk water, and the N-terminus is in a more aqueous-like environment; the pK_a_ reflects this as it increases with larger vesicle size (Figures S7 and S8).

### 2.2. ^1^H NMR Spectroscopy of Diglycine (GG) in RM

**Figure 3:**
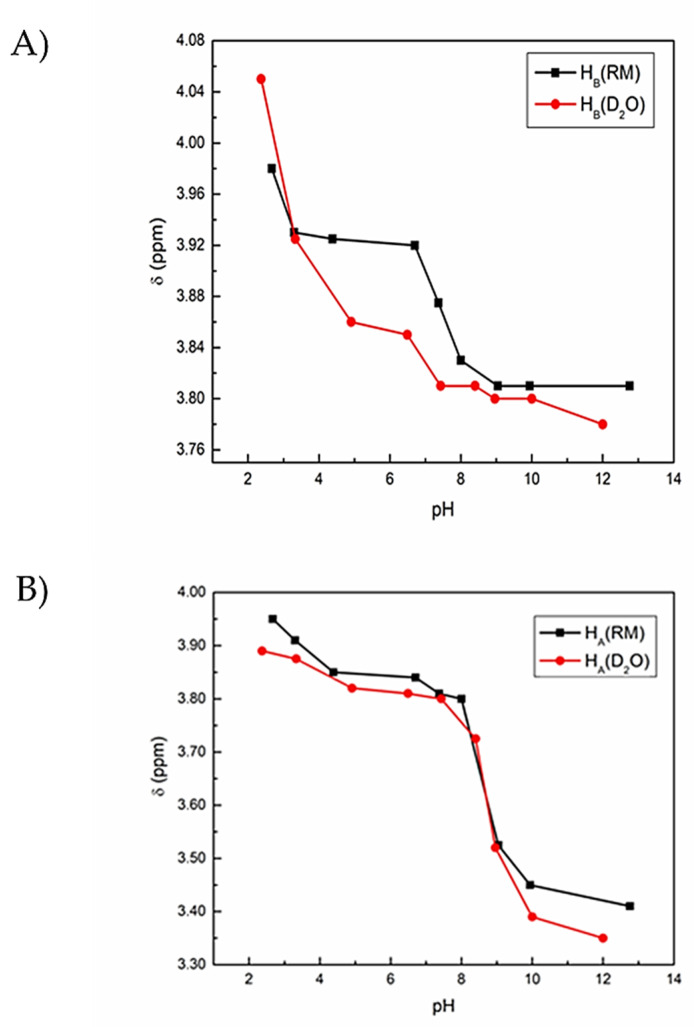
Chemical shifts of GG as a function of pH in D_2_O and RM samples based on ^1^H NMR spectroscopic studies of GG at varying pH values. RMs of size *w_0_* 10 were formed with 200 mM solutions of GG in D_2_O. Error bars on the graph are smaller than the symbols used. (**A**) ^1^H NMR chemical shift values of protons B (CH_2_ near N-terminus) of GG measured at different pH values in D_2_O, with the proton labeling scheme shown in Figure 1C. (**B**) ^1^H NMR chemical shift values of protons A (CH_2_ near C-terminus) of GG measured at different pH values in RMs, with the proton labeling scheme shown in 1C.

In a similar fashion to G, ^1^H NMR spectroscopy of solutions containing GG in the RM model membrane system and aqueous solution was recorded and analyzed to identify any differences in chemical shift that may occur as a result of confinement by *w_0_* 10 RM. Chemical shift values are plotted and compared between environments, with representative spectra for each given in the Supplementary Materials (Figure 3; Figures S9–S12).

The solution pH values and resulting pK_a_ that was calculated show that GG displays a small increase in chemical shift from aqueous environment to the RM, indicating that the compound is in a slightly more charged environment consistent with the interfacial water layer containing the Na^+^ counterions (Figure 1A). However, this change in pK_a_ values from aqueous to RM is small, with a pK_a_ of 2.85 in D_2_O and 2.99 in RM for the C-terminal CH_2_, and 8.60 in D_2_O and 8.48 for the N-terminal CH_2_.

### 2.3. ^1^H NMR Spectroscopy of Triglycine (GGG) in RM

**Figure 4:**
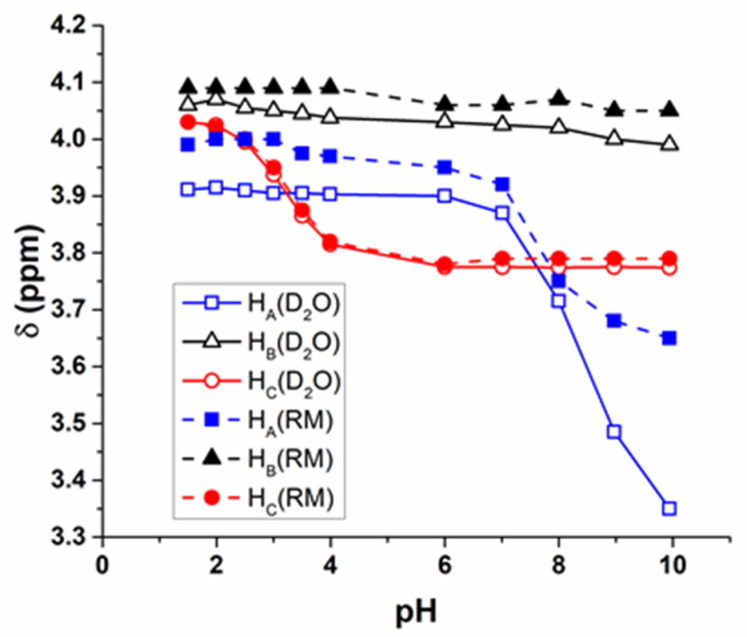
GGG proton shifts compared at varying pH values in aqueous (D_2_O) and RM environments, as determined by ^1^H NMR spectroscopy. RMs of size *w_0_* 10 were formed with 200 mM GGG in D_2_O. Error bars on the graph are smaller than the symbols used. GGG protons are labeled according to Figure 1D.

Solutions containing GGG were also studied in comparison in D_2_O and RMs of *w_0_* 10 using ^1^H NMR to investigate its potential interactions within the confines of the RM. Results obtained from solutions of GGG are similar to those obtained from GG in that there is little change in the chemical shifts of the solutions in aqueous environment and in the AOT RM (Figure 4). There was little change in the pK_a_ of the N- and C-terminal ends of the peptide, with the C-terminal pK_a_ in D_2_O at 3.18, and in RM, 3.27, and the N-terminal pK_a_ in D_2_O was at 8.29, and in RM, 8.11.

We further explored this observation that the chemical shift of the C-terminal CH_2_ remains relatively unchanged between aqueous and RM environments, consistent with the interpretation that the C-terminal end of the peptide resides within the bulk water pool of the RM or the molecule has folded over on itself. The chemical shift of the middle CH_2_ at all pH values tested was slightly elevated in the RM as compared to D_2_O, consistent with being located in a more charged environment, and the N-terminal CH_2_ protons show the most change in chemical shift, with values in the RM being higher than those of D_2_O, consistent with being located in a more charged environment or, possibly, if it is in a folded conformation (Figure 4; Figures S13–S16). However, similarly to those calculated for GG, there is little change in the calculated pK_a_ values with differences of only 0.1 pH unit.

### 2.4. ^1^H NMR Spectroscopy of Tetraglycine (GGGG) in RM

**Figure 5:**
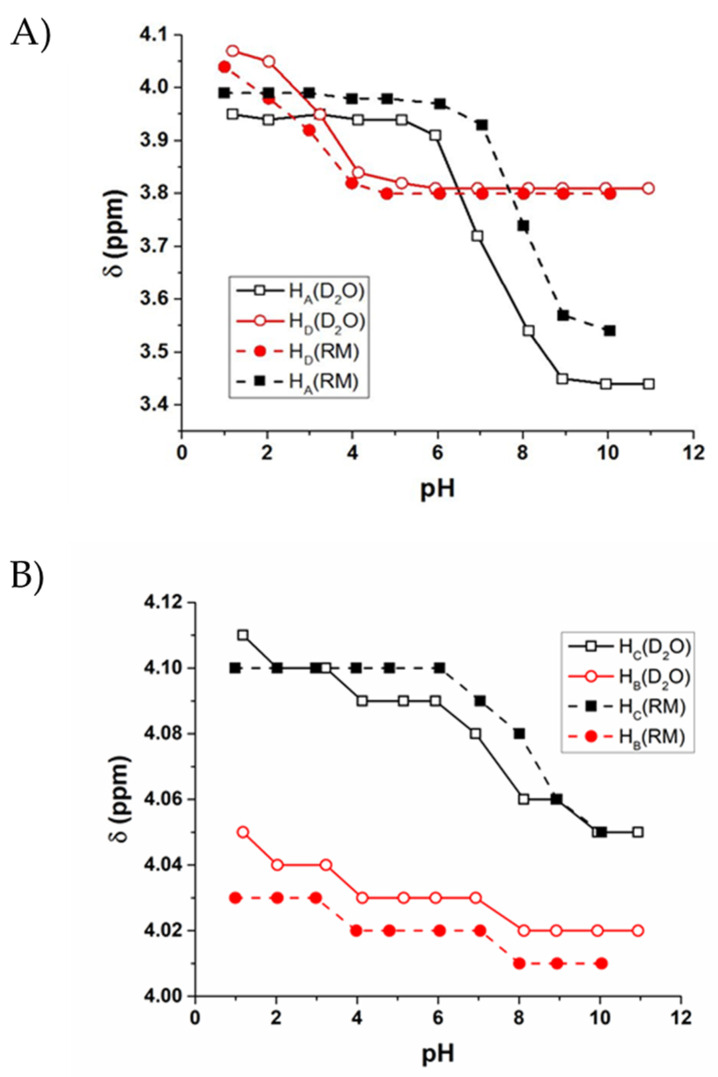
Chemical shift values as determined by ^1^H NMR of GGGG at varying pH values in RM and D_2_O with peaks corresponding to labels in Figure 1D. RMs of size *w_0_* 10 were formed with 200 mM GGGG in D_2_O. Error bars on graph are smaller than symbols used. (**A**) N- and C- terminal CH_2_ protons of GGGG in D_2_O and RM, or protons A and D as labeled in Figure 1E. (**B**) Interior CH_2_ protons on N- or C-terminal side of GGGG in D_2_O and RM, or protons B and C as labeled in Figure 1E.

Aqueous solutions of GGGG at varying pH values and corresponding AOT RMs were analyzed via ^1^H NMR spectroscopy, similarly to the other G compounds above. The results obtained from GGGG in terms of pK_a_ differences are small, as was found for the GGG and GG peptides. The pK_a_ value found for the C-terminal end of the peptide in D_2_O was determined to be 3.05, and that in RM was determined to be 2.82. The pK_a_ value found for the N-terminal end of GGGG was found to be 7.75 in D_2_O and 7.94 within the RM. These small differences may be attributed to the slight changes in the environment of the RM as compared to aqueous solution and suggest that the peptide itself resides between the interface of the RM and the stern layer. The increased pK_a_ of the N-terminal protons as well as the slightly decreased pK_a_ of the C-terminal protons indicate that the zwitterionic form of GGGG is equally or more stable in the RM, which is also consistent with the compound being between the bulk water and interface of the RM (Figure 5A; Figures S17–S20).

It is also worth noting, when looking at the chemical shifts of the middle protons (H_B_ and H_C_) of the compound, the difference in shift is ≤0.1 ppm, indicating that the environment is essentially the same between the two systems (Figure 5B).

### 2.5. Dynamic Light Scattering of RM Samples

To verify that RMs formed in the microemulsion samples, the solutions were subjected to DLS analysis. RMs of sizes of *w_0_* 20 were made as representatives for these investigations instead of the *w_0_* 10 RMs used to perform the ^1^H NMR measurements, as it is much easier both to measure the size as well as to visualize changes with the larger (8.9 nm) *w_0_* 20 RM than *w_0_* 10 (6.8 nm) [35]. The results are summarized in Table 1. Measurements were taken for each solution of RM containing 200 mM G compounds in deionized water (diH_2_O) and the corresponding RM sample with no probe molecule in the diH_2_O. As seen in Table 1, in the larger *w_0_* 20 RM, to better visualize any changes, the size of the RMs did not significantly change by the addition of G, GG, GGG, or GGGG and the values observed are in agreement with the literature value of 8.9 nm for a *w_0_* 20 RM [35].

### 2.6. Compression Isotherms of Langmuir Monolayers Containing Glycine

**Figure 6:**
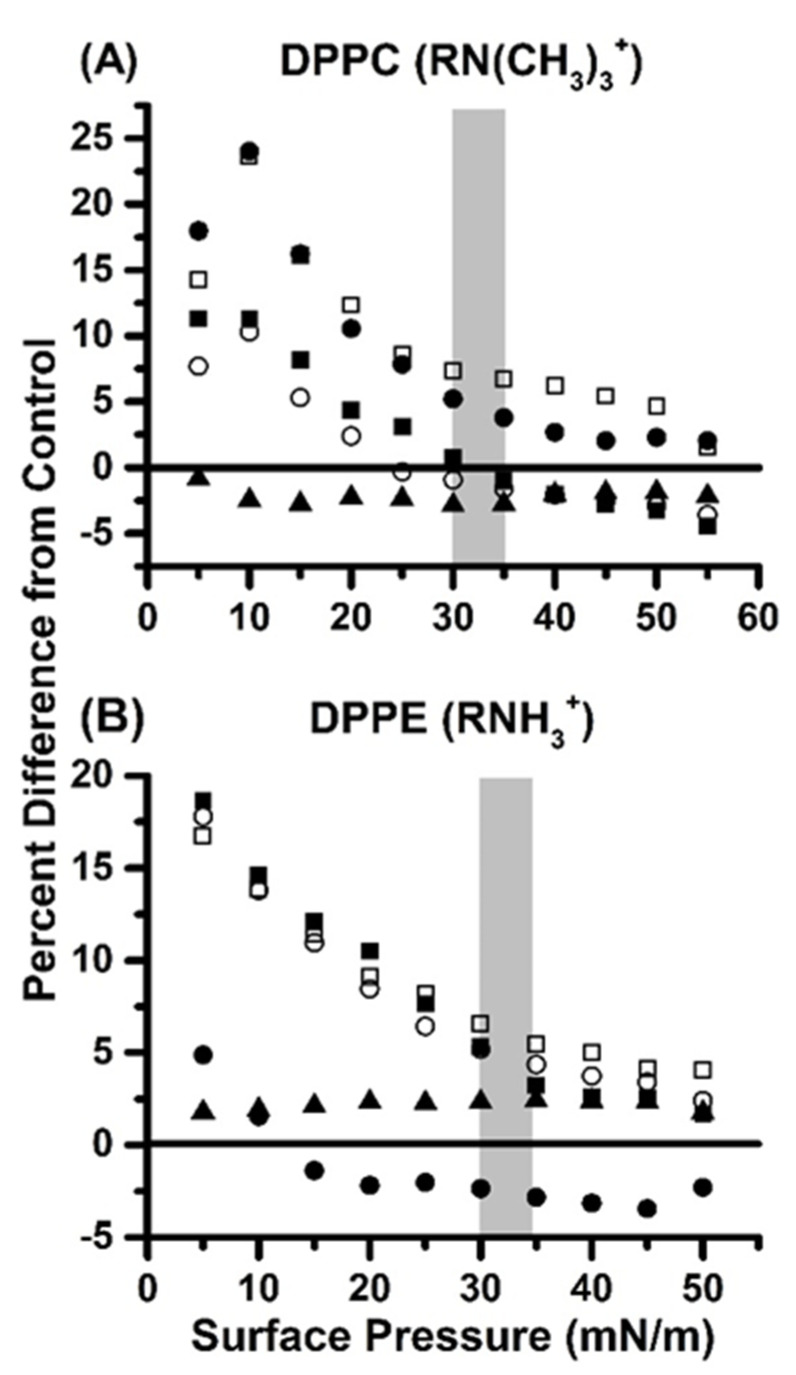
Calculated percent difference between the area per molecule of control Langmuir monolayers and monolayers with glycine in the subphase for (**A**) dipalmitoyl phosphatidylcholine (DPPC) and (**B**) dipalmitoyl ethanolamine (DPPE). R represents the phosphate group, glycerol, and saturated C_16_ tails. Symbols each represent a different pH, where solid squares are pH 4, hollow squares are pH 6, solid circles are pH 7, hollow circles are pH 8, and solid triangles are pH 9. The region shaded in grey represents physiological surface pressure. Exact values and errors for all points are reported in Tables S1 and S2.

In this study, Langmuir monolayers with the lipids dipalmitoyl phosphatidylcholine (DPPC) and dipalmitoyl phosphatidylethanolamine (DPPE), which are two of the most abundant phospholipids found in biological membranes and carry an overall neutral charge, were also used to investigate the effect that glycine has on a biological membrane [46]. Compression isotherm data are plotted as the percent difference in the area per molecule of monolayers containing both lipid and glycine from those containing no glycine versus the surface pressure, as shown in Figure 6 [47]. At pH 4, 6, 7, and 8, DPPC monolayers containing glycine all exhibit a similar trend in which monolayers with glycine present have an expanded area at low surface pressure, but the amount of expansion decreases as surface pressure increases.

However, at pH 6 and 7, monolayers exposed to glycine always remain at least slightly expanded from the control. At pH 4, monolayers with glycine transition from expanded to contracted around 30–35 mN/m, which is what is commonly regarded as physiological surface pressure [48,49]. The pH 8 monolayer with glycine transitioned from expanded to condensed around 25 mN/m. The pH 9 monolayer with glycine in the subphase remained relatively near to the control monolayer at all pressures, though slightly condensed. Importantly, at physiological-like conditions at pH 7 with glycine in the subphase and DPPC as the lipid, the monolayer was 4–5% expanded relative to the control, implying that some glycine was positioned at the interface, as opposed to the subphase or the acyl chains of the DPPC. Overall, DPPC monolayers with glycine in the subphase have a trend of expanding the monolayer at lower surface pressures and then transition to only a slight expansion, or to condensing the monolayer as surface pressure increases. At pH 7, which is the most physiologically relevant pH used in this study, the monolayer remains expanded relative to the control, which suggests that glycine interacts weakly with the interface (Figure 6A).

DPPE monolayers, then, followed nearly the same trend at pH 4, 6, and 8; all are 15–20% expanded relative to the control at a surface pressure of 5 mN/m and decreased as surface pressure increased. All three monolayers reached an equilibrium of remaining approximately 5% expanded relative to the control at 35 mN/m. Much like with the DPPC monolayers, the pH 9 monolayer remained relatively constant, remining between 1.8% and 2.4% expanded relative to the control throughout compression. While glycine slightly condensed DPPC at pH 9, it slightly expanded DPPE at the same pH. Interestingly, pH 7 differs greatly between DPPC and DPPE. For DPPE, the pH 7 monolayer is 5% expanded relative to the control at 5 mN/m and then becomes condensed between 10 and 15 mN/m. The monolayer remains slightly condensed, and at physiological surface pressure, the monolayer exposed to glycine is approximately 2–3% condensed relative to the control.

Since the measured values are within the calculated error, the glycine-exposed monolayer is not experimentally different from the control. Overall, DPPE monolayers with glycine in the subphase at all pH values but seven follow similar patterns to each other, in which the monolayer is expanded 15–20% at lower surface pressures and decreases to 5% expansion as the surface pressure increases (Figure 6B). Interestingly, at pH 7, the monolayer exposed to glycine becomes slightly condensed and does not follow patterns typical of the other pH values, and the experimental error is such that there is no statistical difference between glycine-exposed and control monolayers. This suggests that glycine may interact with the membrane interface, but it does not do so strongly.

## 3. Discussion

The studies described above determine pK_a_ measurements of G-containing peptides and, in doing so, compare the data of small G-peptides in aqueous solution and associated with the AOT interface. The longer G peptides, in the case of GGG and GGGG, have chemical shifts in the same region as the AOT and overlap in chemical shifts, as can be seen in Figures S15 and S19. G and GG are found to appear in a region where AOT and isooctane peaks are not observed; however, GGG and GGGG show signals in the same region as the AOT, therefore limiting observation of the signals of these short G-containing peptides. As a result, a subtraction method was utilized in which the AOT RM spectra containing no compound were subtracted from AOT RM spectra containing the G compound of interest. Analyzing the spectra using the subtraction method described in the experimental section allowed us to calculate the chemical shifts for all G compounds and was also used to obtain the pK_a_ results, summarized in Table 2. The pK_a_ values were calculated for both the R-group protons near the C-terminus and the N-terminus of the G peptides in both aqueous environment and the environment of the RM. The resulting pK_a_ values calculated in this work are summarized in Table 2 for all the systems investigated in this work and detailed in the descriptions below.

**Figure 7:**
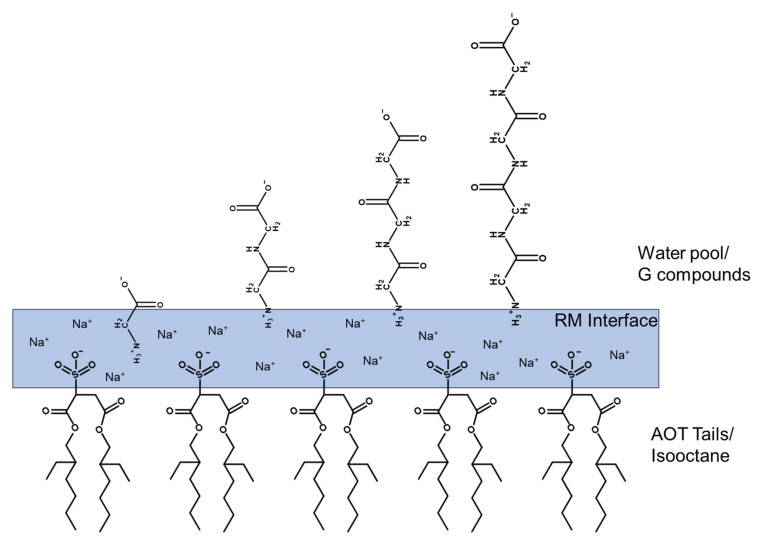
Schematic figure depicting the likely positioning of the G compounds used in this study relative to the RM interface. G compounds depicted here are shown in a linear conformation; however, it is likely that longer G compounds such as GGG and GGGG rotate around C-C bonds in solution such that the conformation of the molecule may be bent as discussed previously, but the C-terminal end is still located at the bulk water pool.

Our hypothesis that G is likely positioned such that the N-terminus is near or in the interstitial water region of the RM either facing the negatively charged interface or actually associated with the interface is in line with previous observations and predictions with other charged molecules [51,52]. This pattern was observed for all the G peptides to different degrees, with the largest change for G. The larger difference for G can be explained because this is a smaller amphiphilic molecule, and penetration of the interface by the N-terminus will impact the amphiphilic molecule more than with peptides. Although penetration will bring the C-terminus closer to the interface, little change is observed in the pK_a_ of the C-terminal, suggesting that the N-terminal is loosely associated with the interface and not deeply penetrated in the interface (Figure 7). This difference in the pK_a_ of the protonated amine of G may also be due to the presence of ions at the RM interface; in pure water, G forms an energetically favorable five-membered ring between the protons of the positively charged amine and the negatively charged oxygen on the carboxyl group, which is disrupted by the presence of ions at the RM interface where the N-terminus is likely located. This will result in a lower pK_a_, as shown in Table 2. The changes in the pK_a_ value of the amino terminus in the three G-peptides, by contrast, are much smaller. Differences in pK_a_ between different sizes of RMs containing G suggest that there may be some subtle differences in the specific location of the amino terminus of these G peptides as anticipated, because the charge distributions are somewhat different depending on the specific conformation of the molecule. Importantly, modest change is observed in the pK_a_ value of the C-terminus consistent with its environment changing much less compared to the aqueous and microemulsion preparations of the G compounds, consistent with their location closer to or in the bulk water pool of the RM as expected if the environment changed little [45].

The presented data for all the G peptides investigated indicate that they all interact with the interface, albeit in different ways. The smallest G, which is a zwitterion at neutral pH, is likely to interact more strongly with the interface based on the large changes in the pK_a_ of the free amine part of the peptide. As we demonstrated with aniline, the observed differences are likely to be caused by changes in location and not due to an inherent difference in pK_a_ values in the new environment [52]. This may also be due to the disruption of the favorable five-membered ring that is formed by the positively charged protons on the amine and the negatively charged oxygen on the carboxylate group in water by the presence of Na^+^ at the RM interface, as mentioned previously. In the case of the GG, GGG, and GGGG peptides, the observed difference is much less and there are also variations in the direction of the change; for G, GG, and GGG, the pK_a_ value decreased (acidity increased) in the presence of the interface, whereas in the case of GGGG, the pK_a_ value increased (acidity decreased). In order to obtain more information on this system, we examined the interactions of glycine with lipid interfaces in the Langmuir monolayer system. Since the majority of responses were observed with glycine, we limited these studies with glycine but examined its response in a pH-dependent manner (Section 2.6). These results showed that glycine is likely to associate with the lipid interface at near-neutral pH, hence confirming the observations made with the microemulsion system.

Comparison of the pK_a_ values in aqueous solution and near RM interfaces is most valuable when considering the inherent differences between the two systems and recognizing that the aqueous solution can change significantly depending on the other ions in solution and overall ionic strength. Previous work done with GGG in aqueous solution found that GGG adopts a U-shaped conformation in the presence of Na^+^ and SO_3_^−^ [53], with no similar studies being found for GG or GGGG. In this study, it was found that there is a strong interaction between the sodium ions and the sulfite, which then interacts with the protonated amine of GGG, favoring a bending that adopts a U shape. A similar phenomenon may be occurring in the RM systems in which the Na^+^ ions interact with the sulfate groups on the AOT surfactant molecules, which then interact more strongly with the protonated amine. This would be consistent with the increase in chemical shift of the protonated amine for GGGG in RM as compared to its shift in D_2_O (Table 2). Additionally, the increased pK_a_ of the N-terminal protons as well as the slightly decreased pK_a_ of C-terminal protons indicates that the zwitterionic form is equally or more stable in the RM, consistent with being at the edge of the bulk water pool of the RM between the bulk water and interface (Figure 5A,B; Figure 7).

These results imply that it is unlikely that peptides containing numerous glycine residues will have a strong effect as membrane-penetrating peptides for use in the development of novel antibacterial or anticancer therapeutics, unless there are other amino acids present which are more likely to interact with a membrane interface, such as lysine. Even in the context of the reverse micelle, which has a strongly negatively charged interface to mimic the exposure of phosphatidylserine residues by cancerous cells, there is little to no interaction of the G peptides with the RM interface, indicating that even when the interface has a negative charge characteristic to bacterial or cancerous cells, there is still no penetration of the interface by a peptide, despite numerous reports of glycine-rich AMPs [26,27,28]. This result stands in contrast with studies with G alone, which is found to interact with the interface. Together, these results suggest that for AMP peptides to be effective in penetrating membranes, residues other than G are necessary for the action of these peptides. This is consistent with the fact that many AMP peptides contain significantly higher concentrations of lysine residues and/or aromatic residues such as phenylalanine and tyrosine in addition to higher concentrations of glycine than the average presence of these amino acids in other proteins due to the two physical features required for antimicrobial peptide activity: charge and hydrophobicity [54,55,56].

## 4. Materials and Methods

### 4.1. General Materials

The following materials were purchased and used without purification: glycine HCl (G, Mallinckrodt, Madison, WI, USA, 99.0%), diglycine (GG, Sigma-Aldrich, St. Louis, MO, USA, 99.0%), triglycine acid (GGG, Sigma-Aldrich, 99.0%), tetraglycine (GGGG, Aurum Pharmatech, Franklin Park, NJ, USA, >96%), activated charcoal (carbon 6–12 mesh), 2,2,4-trimethylpentane (isooctane) (Sigma-Aldrich, 99.0%), deuterium oxide (Sigma-Aldrich, 99.0% deuterium), and 4,4,-dimethyl-4-silapentane-1-sulfonic acid sodium salt (DSS, Wilmad, Buena, NJ, USA). The chemicals methanol (>99%), citric acid anhydrous (>99.5%), sodium citrate dihydrate (>99%), sodium hydroxide (>99%), and hydrochloric acid were purchased from Fisher Scientific. The lipids 1,2-dipalmitoyl-*sn*-glycero-3-phosphocholine (DPPC, >99%) and 1,2-dipalmitoyl-*sn*-glycero-3-phosphoethanolamine (DPPE, >99%) were purchased from Avanti Polar Lipids (Alabaster, AL, USA). Sodium Aerosol-OT (AOT) (bis(2-ethylhexyl)sulfosuccinate sodium salt, Sigma-Aldrich, ≥99.0%) was purified as described previously to remove an acidic impurity [57]. Briefly, 50.0 g of AOT was dissolved into 150 mL of methanol to which 15 g of activated charcoal was added. This suspension was stirred for 2 weeks. After mixing, the suspension was filtered to remove the activated charcoal. The filtrate was then dried under rotary evaporation at 50 °C until the water content was below 0.2 molecules of water per AOT as determined by ^1^H NMR spectroscopy [58]. The pH of aqueous solutions was measured at 25 °C on an Orion 2STAR pH meter (Thermo Fisher Scientific, Waltham, MA, USA) prior to formation of the AOT RM in isooctane. The pH was adjusted throughout the experiment using varying concentrations of NaOH or HCl dissolved in diH_2_O or D_2_O, depending on experimental need. NaOH or HCl dissolved in D_2_O is referred to as NaOD or DCl, respectively, and the pH was adjusted to consider the presence of deuterium (pD = 0.4 + pH) [58,59]. The pD is customarily referred to as pH and will be referenced as such for the remainder of this manuscript.

### 4.2. Preparation of Samples for Analysis

#### 4.2.1. Preparation of Stock Solutions of G, GG, GGG and GGGG for ^1^H NMR and Dynamic Light Scattering

Each of the 200 mM stock solutions used in the ^1^H NMR experiments were prepared with 2.00 × 10^−3^ mol each of G, GG, GGG, and GGGG dissolved in 10 mL D_2_O in a volumetric flask and pH-adjusted to the appropriate value as needed for the overall concentration of 200 mM. Each of the 50 mM stock solutions used for dynamic light scattering experiments were prepared with 5.0 × 10^−3^ mol each of G, GG, GGG, and GGGG and dissolved in 10 mL diH_2_O.

All stock solutions were sonicated until clear, if not already, and all stock solutions were pH-adjusted with DCl or HCl and NaOD or NaOH, depending on experimental need. The pH of the stock solutions was measured at 25 °C with an Orion 2STAR pH meter. The pH values were measured directly in D_2_O, and the pH was adjusted to the presence of deuterium (pD) and is referred to as pH rather than pD, as stated previously [59,60,61].

#### 4.2.2. Preparation of AOT-Isooctane Stock Solution and RMs Containing G, GG, GGG, and GGGG for ^1^H NMR

A 750 mM AOT-isooctane stock solution was prepared by dissolving 7.5 × 10^−3^ mol AOT in 10 mL isooctane. This mixture was sonicated and vortexed until clear, approximately 15 min. Once dissolved, the solution was equilibrated to ambient room temperature. RMs of *w_0_* values of 6, 10, 14, 16, and 20, where *w_0_* = [H_2_O]/[AOT], were prepared by combining appropriate volumes of the appropriate prepared stock AOT stock solution, depending on experimental need, and appropriate volumes of 200 mM stock solutions of G, GG, GGG, or GGGG to create the desired size of RM. 

#### 4.2.3. Preparation of AOT-Isooctane Stock Solution and RMs Containing G, GG, GGG, and GGGG for Dynamic Light Scattering

The 200 mM AOT-isooctane solution was prepared by dissolving 2.00 × 10^−3^ mol AOT in 10 mL isooctane. This mixture was sonicated and vortexed until clear, approximately 15 min. Once dissolved, the mixture was equilibrated to ambient room temperature. To prepare the RM solutions, specific volumes of AOT stock solution and aqueous 50 mM G stock solution were combined to a total of 5 mL to form RM sizes of *w_0_* 10 and 20 (*w_0_* = [H_2_O]/[AOT]). This mixture was vortexed until clear, consistent with the formation of RMs.

#### 4.2.4. Preparation of Lipid Stock Solutions and Aqueous Subphase

Sodium phosphate buffer (20 mM) was prepared in distilled deionized water and adjusted to pH 6.00, 7.00, 8.00, and 9.00 (±0.02) with either 1.0 M HCl or 1.0 M NaOH. Sodium phosphate citrate buffer (20 mM) was prepared in distilled deionized water and adjusted to pH 4.00 ± 0.02 in the same manner as the sodium phosphate buffers. Glycine subphase (1 mM) was prepared by dissolving 75.0 ± 0.1 mg glycine into one liter of the previously prepared buffers. The pH was readjusted to the previously mentioned values with 1.0 M HCl or 1.0 M NaOH. Stock solutions of DPPC and DPPE were prepared by dissolving 0.025 mmol of powdered phospholipid into 5.0 ± 0.1 mL of freshly prepared 9:1 chloroform methanol (v:v).

### 4.3. Methods

#### 4.3.1. ^1^H NMR Spectroscopy and Analysis of D_2_O and RM Samples

The ^1^H NMR experiments were performed using a 400 MHz Varian (Gloucester, MA, USA) ^1^H NMR spectrometer using standard parameters (1 s relaxation time, 25 °C temperature control, and 45° pulse angle). The aqueous samples were referenced to an internal DSS sample. RM samples were referenced to the isooctane methyl peak at δ = 0.90 ppm as has been previously reported and were originally referenced to tetramethylsilane [51]. The resulting spectra were referenced, baseline-corrected, normalized, and analyzed using MestReNova version 10.0.1.

The pK_a_ values were determined by plotting chemical shifts of the samples at their varying pH values in D_2_O and *w_0_* 10 RMs and calculating the first derivative of the best fit curve using OriginPro version 9.1 [62]. In order to do this, a plot was made of ppm vs. pH and the curve was fitted in Origin using the reference described in [62] for monofunctional acids. In order to do this, half of the bifunctional curve of ppm vs. pH was plotted and a best fit line was applied. From here, the first derivative was calculated to give the final pK_a_ value for both the carboxyl- and amine-terminal protons (Figure S2). 

**Figure 8:**
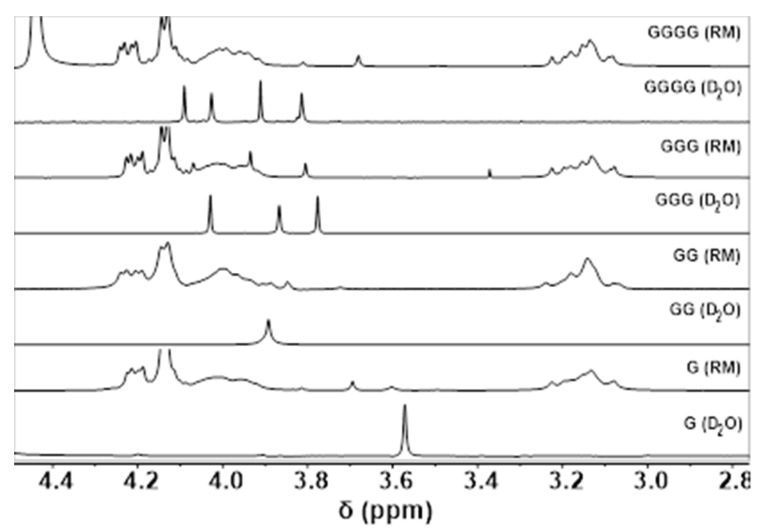
A comparison of each of the studied compounds and their chemical shifts as determined by ^1^H NMR in *w_0_* 10 reverse micelles (RMs) and D_2_O. Spectra beginning from the bottom correspond to G, GG, GGG, and GGGG in D_2_O and RM alternately at pH 7. Asterisk in the GGG RM spectrum denotes acetone impurity.

In the case of peaks corresponding to GGG and GGGG in RMs, these shifts were often masked by the AOT peaks, as shown in Figure 8. As a result, a technique was employed in which worked up spectra were analyzed by MestReNova version 10.0.1, and after baseline correction, normalization, and referencing, the arithmetic function in MestReNova was used to subtract control spectra containing no probe molecule from spectra which did contain probe molecules (Figures S15 and S19).

#### 4.3.2. Langmuir Trough Instrument Preparation

Compression isotherms were obtained with a Kibron μTrough XS (stainless steel; Helsinki, Finland) equipped with a hydrophobic Teflon ribbon barrier. The trough was cleaned thoroughly with three isopropanol washes, three ethanol washes, and a water rinse before each experiment. Excess water was evaporated with compressed air. The wire probe used as a Wilhelmy plate was flamed with a Bunsen burner to remove lipids before each experiment.

After cleaning, approximately 50 mL of 20 mM buffer or 1 mM glycine in 20 mM buffer was added to the trough. The subphase surface was then cleaned with vacuum aspiration to remove dust contamination. The surface was considered clean when the surface pressure remained at 0.0 ± 0.5 mN/m throughout a full compression.

#### 4.3.3. Formation, Compression Measurement, and Calculation of Langmuir Monolayers

Either DPPC or DPPE (20 µL, 20 nmol) was added to the surface in a drop-wise manner with a glass Hamilton syringe (50 µL) followed by a 15-min equilibration period. Monolayers were compressed at a speed of 10 mm/min (5 mm/min on each side). The temperature of the subphase was maintained at 25 °C by an external water bath. All experiments were run in triplicate, and the data presented were obtained by averaging the triplicate measurements.

The percent difference between control monolayers and monolayers with glycine present in the subphase was calculated with Equation (1), where *A_gly_* is the area of monolayers with glycine present and *A_con_* is the area of control monolayers. Calculations were performed at every 5 mN/m of surface pressure.

(1) $$\%diff=\left(\frac{A_{gly}-A_{con}}{A_{con}}\right)\times 100$$

#### 4.3.4. Dynamic Light Scattering (DLS)

Dynamic light scattering (DLS) experiments were performed using the Malvern Instrument (Malvern Instruments Limited, UK) MAN0486 [36,51]. DLS and the autocorrelation method of analyzing scattering were used to measure the hydrodynamic radius of AOT RMs, with temperature controlled at 25.0 °C. Each sample was equilibrated for 600 s at 25 °C and then run for 10 scans per acquisition for a minimum of ten measurements for every solution, with and without G compounds, at neutral pH (7.4) for each *w_0_* value. A 1-mL aliquot of sample was required for measurement. The viscosity (0.4670 cP) and refractive index (1.391) were needed for RM size determination in the isooctane solvent used in this work [57]. The photons scattered by the RMs were collected at a 173° angle. Data processing was carried out using the Zetasizer version 7.11 software.

## 5. Conclusions

Studies exploring the interaction of G, GG, GGG, and GGGG compounds with model membrane interfaces measured in microemulsions (AOT RMs) using ^1^H NMR spectroscopy and DLS indicate that G peptides prefer to locate themselves at the edge of the charged reverse micellar interface, between the water pool and interface at the stern layer. This location is different for the single amino acid G, which is penetrated further into the interface. These findings are supported by the calculated pK_a_ values of the G compounds in both aqueous and RM systems. Minor differences were observed for the pK_a_ values and the chemical shift between the aqueous and micellar environments, indicating similarity between the environments that the G peptides are inhabiting. Larger changes were observed for the amine group on the G amino acid, suggesting that the N-terminus is further anchored into the interface. This finding is also consistent with studies done with Langmuir monolayers containing DPPE and DPPC exposed to glycine; in the case of DPPC, at physiological pH, the interface remains only slightly expanded relative to the control, indicating weak interaction with the interface. At physiological pH, there was no significant difference between DPPE monolayers exposed to glycine and the control. In the case of the short G peptides GG, GGG, and GGGG, it is likely that they associate with the RM interface by orienting themselves such that the N-termini interact weakly with the RM interface and the C-termini oriented towards the bulk water pool of the RM (Figure 7).

The case of G is very different from that of its longer peptides. In an aqueous environment, the protons on the positively charged amine hydrogen bond with the negatively charged carboxyl end and form an energetically favorable five-membered ring. This may explain the large difference between the pK_a_ measured in the aqueous environment compared to the reverse micellar environment. In the RM, this hydrogen bonding is disrupted, likely by the presence of the Na^+^ counterions. Additionally, the observed gradually decreasing chemical shift of G at pH 7 and 9 indicates that the amino acid is likely placed in the interstitial water layer between the interface and the bulk water pool. As the RM grows larger and the water becomes more similar to bulk water, the chemical shift approaches a shift more analogous to that in D_2_O, consistent with the G moving from the interface into the interior water pool. This conclusion is very important because of the role of G as a neurotransmitter; that is, for G to function and propagate a signal to be received after it has been confined within synaptic vesicles and excreted through the synapse. These results suggest that in the large synaptic vesicle (40 nm), it is not likely that G will have any significant interactions with the membrane interface and is readily released for uptake [63].

Considering that AMPs (host defense peptides) generally contain a high level of G as well as other key amino acids (Lys, Phe/Tyr) it was of interest to determine the effects of G and G peptides to obtain a better understanding how specific amino acid residues and their corresponding peptides interact with membranes. The data suggest that the amino acid G does associate with the membrane whereas the G peptides interact less strongly with a membrane and likely function to increase the hydrophobicity of reported AMPs which are glycine-rich. These studies support the interpretation that the properties of AMP peptides are more related to other amino acids such as Lys and aromatic amino acids with regard to translocation of these peptides across a membrane for anticancer or antimicrobial activities.

## Figures and Tables

**Table 1:** Dynamic light scattering (DLS) size measurements of RM containing each of the G peptides. Each of these measurements was taken at pH 7.

Sample	*w_0_* 20 RM Diameter (nm)	*w_0_* 20 RM Std. Dev. (nm)
Control	9.5	0.44
G	9.5	0.43
GG	9.2	0.47
GGG	9.2	0.36
GGGG	9.3	0.39

**Table 2:** Comparison of experimental pK_a_ values obtained for G compounds in aqueous (D_2_O) and *w_0_* 10 RM systems, shown with 95% confidence intervals, with literature aqueous pK_a_ values.

Compound	System	pK_a_(1): This Work, Carboxylic Acid	pK_a_(1) lit.	pK_a_(2): This Work, Protonated Amine	pK_a_(2) lit.
G	D_2_O	2.51 ± 0.03	2.46 [50]	10.7 ± 0.05	9.60 [50]
	RM	2.49 ± 0.02		8.51 ± 0.06	
GG	D_2_O	2.85 ± 0.08	3.15 [50]	8.60 ± 0.10	8.10 [50]
	RM	2.99 ± 0.04		8.48 ± 0.04	
GGG	D_2_O	3.18 ± 0.03	3.18 [50]	8.29 ± 0.04	7.87 [50]
	RM	3.27 ± 0.02		8.11 ± 0.07	
GGGG	D_2_O	3.05 ± 0.08	3.25 [50]	7.75 ± 0.12	7.98 [50]
	RM	2.82 ± 0.09		7.94 ± 0.14	

## Supplementary Materials

Supplementary materials can be found at https://www.mdpi.com/1422-0067/22/1/162/s1. Figure S1. NMR spectra documenting the insolubility of glycine (G) in isooctane; Figures S2–S4. ^1^H NMR spectra at different pH values of G in D_2_O, a plot of ^1^H NMR chemical shifts as a function of pH of G in D_2_O and a representative calculation of the pK_a_ of the bifunctional amino acids/peptides used in this manuscript.; Figures S5 and S6. ^1^H NMR spectra at different pH values of G in RM and plot of ^1^H NMR chemical shifts as a function of pH of G in RM; Figures S7 and S8. ^1^H NMR spectra at different pH values of G in *w_0_* 30 RM and a plot of ^1^H NMR chemical shifts as a function of pH of G in *w_0_* 30 RM; Figures S9 and S10. ^1^H NMR spectra at different pH values of diglycine (GG) in D_2_O and a plot of ^1^H NMR chemical shifts as a function of pH of GG in D_2_O; Figures S11 and S12. ^1^H NMR spectra at different pH values of GG in RM and a plot of ^1^H NMR chemical shifts as a function of pH of GG in RM; Figures S13 and S14. ^1^H NMR spectra at different pH values of triglycine (GGG) in D_2_O and a plot of ^1^H NMR chemical shifts as a function of pH of GGG in D_2_O; Figures S15 and S16. Subtraction example of ^1^H NMR spectrum of GGG in RM and plot of ^1^H NMR chemical shifts as a function of pH of GGG in RM; Figures S17 and S18. ^1^H NMR spectra at different pH values of tetraglycine (GGGG) in RM and a plot of ^1^H NMR chemical shifts as a function of pH of GGGG in D_2_O; Figures S19 and S20. Subtraction example of ^1^H NMR spectrum of GGGG in RM and a plot of ^1^H NMR chemical shifts as a function of pH of GGGG in RM; Table S1 Percent difference values for DPPC-Glycine Langmuir monolayers; Table S2. Percent difference values for DPPE-Glycine Langmuir monolayers.

## Abbreviations

AMPs	Antimicrobial peptides, also known as host defense peptides
AOT	Aerosol-OT
DLS	Dynamic Light Scattering
DPPC	Dipalmitoyl phosphatidylcholine
DPPE	Dipalmitoyl phosphatidylethanolamine
G	Glycine
GG	Diglycine
GGG	Triglycine
GGGG	Tetraglycine
NMR	Nuclear Magnetic Resonance
RMs	Reverse Micelles
