# Supplementary material for: The Interfacial Interactions of Glycine and Short Glycine Peptides in Model Membrane Systems
Source: Int J Mol Sci. 2020 Dec 26;22(1):162. doi: 10.3390/ijms22010162 (PMC7795424; doi:10.3390/ijms22010162)
Supplement: Supplementary file 1 [file ijms-22-00162-s001.pdf]

The Interfacial Interactions of Glycine and Short Glycine Peptides in Confined Spaces

Kaitlin A. Doucette[a,b], Nuttaporn Samart[a,d], Prangthong Chaiyasit[a,c], Donn Calkins[a], Callan A. Knebel[a], Kayli N. Martinez[a], Cameron van Cleave [a], Sreekanth Thota[a], and Anan Tongraar[c] and Debbie C. Crans*[a,b]

[a]Department of Chemistry Colorado State University, Fort Collins, Colorado 80523, USA

[b]Cell & Molecular Biology Program, Colorado State University, Fort Collins, Colorado 80523, USA

[c]School of Chemistry, Institute of Science, Suranaree University of Technology, Nakhon Ratchasima, 30000 (Thailand)

[d]Department of Chemistry, Rajabhat Rajanagarindra University, Chachoengsao 24000, Thailand

Table of Contents

Figure S1. NMR spectra documenting the insolubility of glycine (G) in isooctane

Figure S2. Representative calculation of the pK_a of the difunctional acids used in this manuscript.

Figure S3. ^{1}H NMR spectra at different pH values of G in D_2O

Figure S4. A plot of ^{1}H NMR chemical shifts as a function of pH of G in D_2O

Figure S5. ^{1}H NMR spectra at different pH values of G in RM

Figure S6. A plot of ^{1}H NMR chemical shifts as a function of pH of G in RM

Figure S7. ^{1}H NMR spectra at different pH values of G in w_0 30 RM

Figure S8. A plot of ^{1}H NMR chemical shifts as a function of pH of of G in w_0 30 RM

Figure S9. ^{1}H NMR spectra at different pH values of diglycine (GG) in D_2O

Figure S10. A plot of ^{1}H NMR chemical shifts as a function of pH of GG in D_2O

Figure S11. ^{1}H NMR spectra at different pH values of GG in RM

Figure S12. A plot of ^{1}H NMR chemical shifts as a function of pH of GG in RM

Figure S13. ^{1}H NMR spectra at different pH values of triglycine (GGG) in D_2O

Figure S14. A plot of ^{1}H NMR chemical shifts as a function of pH of GGG in D_2O

Figure S15. Subtraction example of ^{1}H NMR spectrum of GGG in RM

Figure S16. A plot of ^{1}H NMR chemical shifts as a function of pH of GGG in RM

Figure S17. ^{1}H NMR spectra at different pH values of tetraglycine (GGGG) in RM

Figure S18. A plot of ^{1}H NMR chemical shifts as a function of pH of GGGG in D_2O

Figure S19. Subtraction example of ^{1}H NMR spectrum of GGGG in RM

Figure S20. A plot of ^{1}H NMR chemical shifts as a function of pH of GGGG in RM

Table S1. Percent difference values for DPPC-Glycine Langmuir monolayers

Table S2. Percent difference values for DPPE-Glycine Langmuir monolayers

NMR spectra documenting the insolubility of glycine (G) in isooctane:

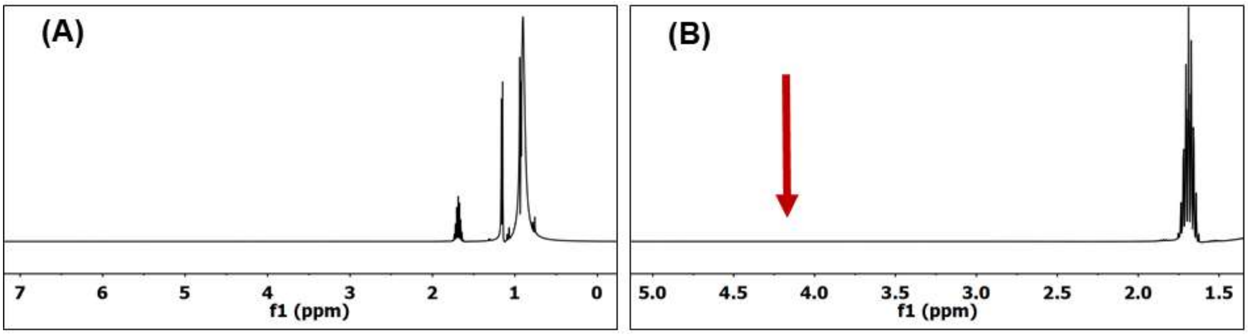

Figure S1. ^{1}H NMR spectra of isooctane with glycine taken after 48 hours. The spectra were referenced to the isooctane peak at 0.904 ppm. (A) shows the spectrum from 0 to 7 ppm. (B) shows the same spectrum from 1.5 to 5 ppm. Glycine is expected to have peaks around 4.3 ppm which are not present, showing that glycine does not dissolve in isooctane consistent with the charged nature of the amino acid.

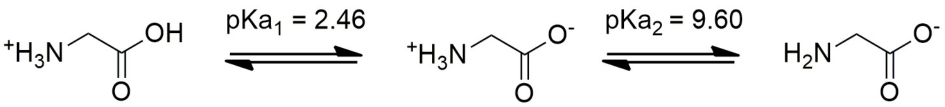

Figure S2. Representative calculation of the pKa of the difunctional acids used in this manuscript. (A) shows the best fit line generated by the chemical shift data obtained from G at varying pH values for the carboxylate group and (B) shows the first derivative of this curve. (C) then shows the best fit line generated by the chemical shift data obtained from G at varying pH values for the amine group and (D) shows the first derivative of this curve.

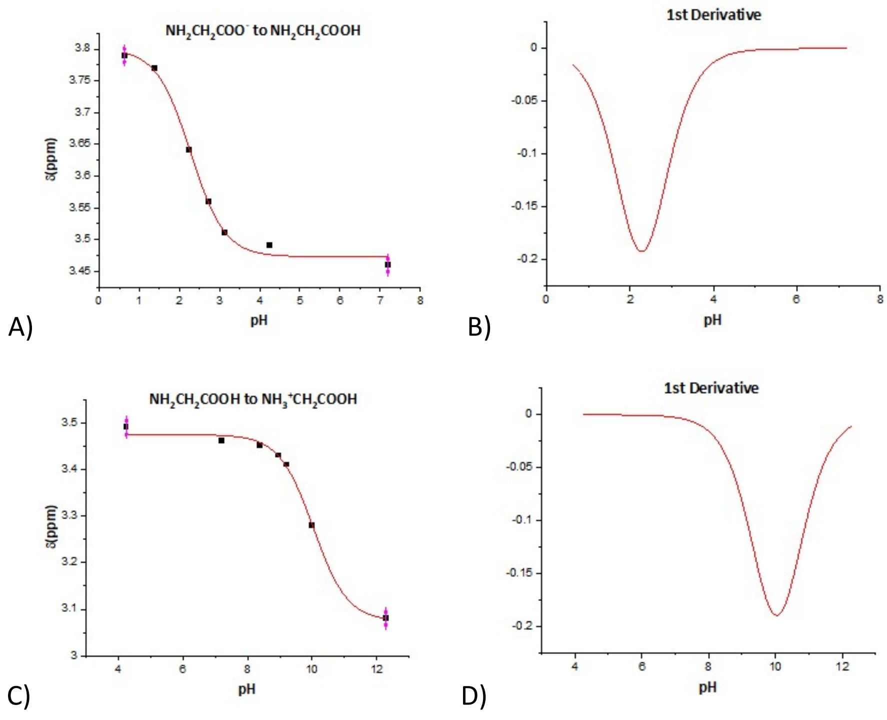

Figure S3. ^{1}H NMR (400 MHz, Bruker) of deuterium oxide solutions of glycine (200 mM) are shown at various pH values. Samples are referenced externally against DSS. Spectra were run in triplicate and one representative series is shown.

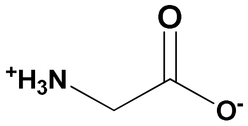

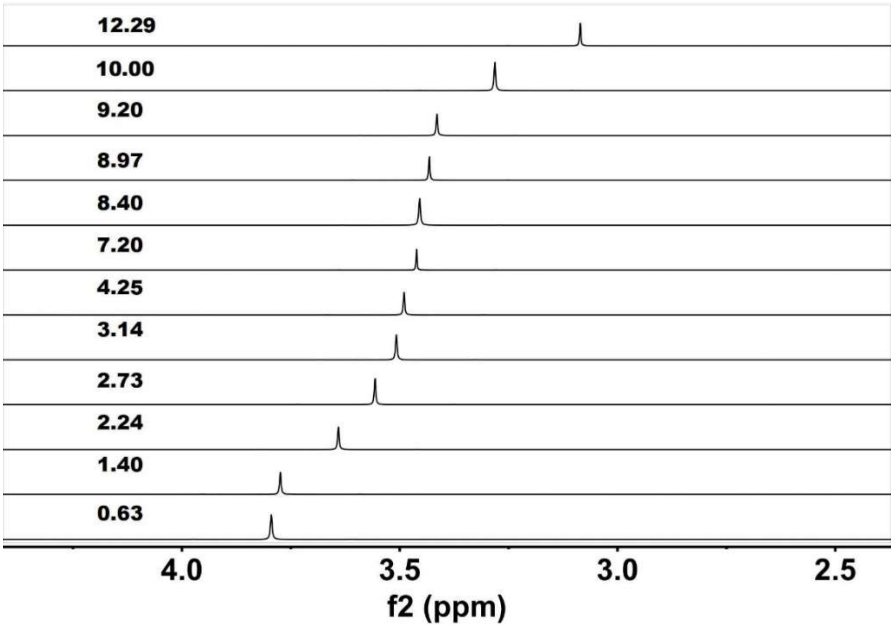

Figure S4. Chemical shifts listed for the ^{1}H NMR spectrum glycine (G) at different pH (left), as well as a plot of ^{1}H NMR chemical shifts as a function of pH (right). Chemical shifts are obtained from the data shown in Figure S2.

pH	Chemical shift (δ) assignment (Ha)
0.63	3.79±0.002
1.40	3.77±0.001
2.24	3.64±0.003
2.73	3.56±0.002
3.14	3.51±0.005
4.25	3.49±0.003
7.20	3.46±0.002
8.40	3.45±0.002
8.97	3.43±0.001
9.20	3.41±0.005
10.00	3.28±0.002
12.29	3.08±0.001

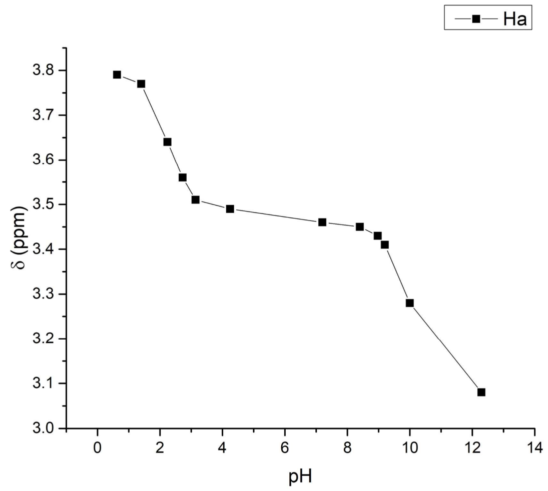

Figure S5. ^{1}H NMR (400 MHz, Bruker) of deuterium oxide solutions of glycine (200 mM) are shown at various pH values added to 750 mM AOT/isooctane in *w*$_0$10 RMs. Spectra were run in triplicate and one representative series is shown. Glycine peaks are denoted by asterisks.

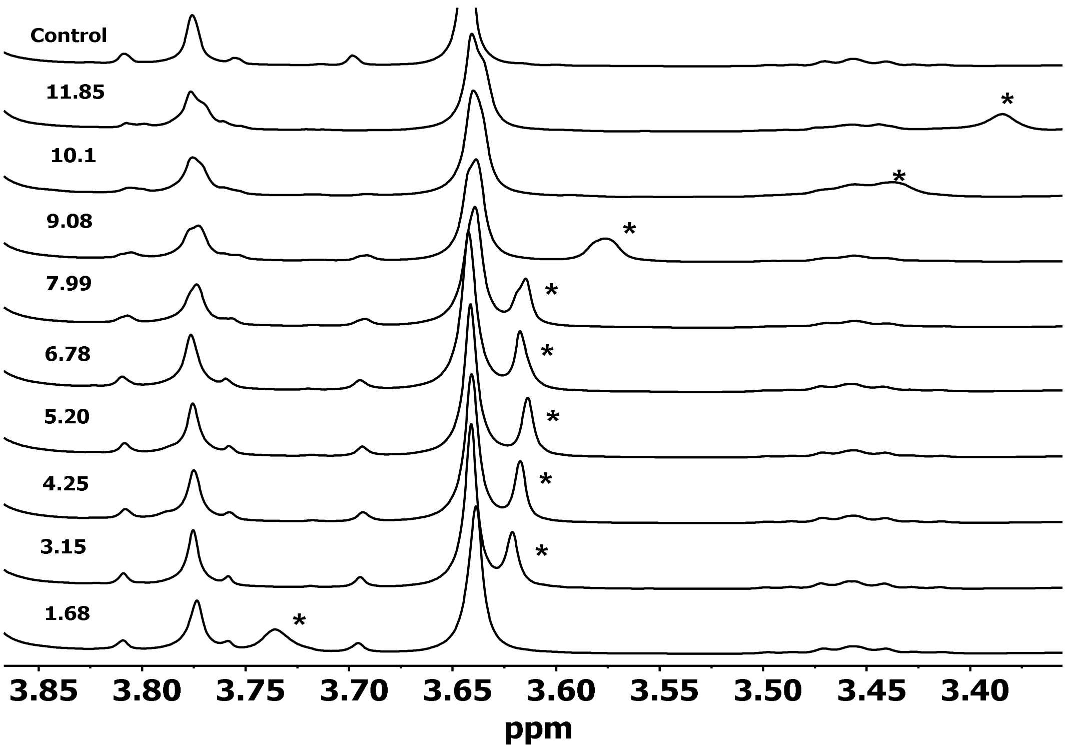

Figure S6. pH-dependent Chemical shifts listed for the ^{1}H NMR spectrum of glycine (G) in RM (left), and a plot of ^{1}H NMR chemical shifts as a function of pH in RM (right). Chemical shifts are obtained from the data shown in Figure S4.

pH	Chemical shift (δ) assignment
1.68	3.74±0.001
3.15	3.62±0.007
4.25	3.62±0.003
5.20	3.61±0.001
6.78	3.61±0.004
7.99	3.61±0.003
9.08	3.58±0.002
10.10	3.44±0.001
11.85	3.38±0.004

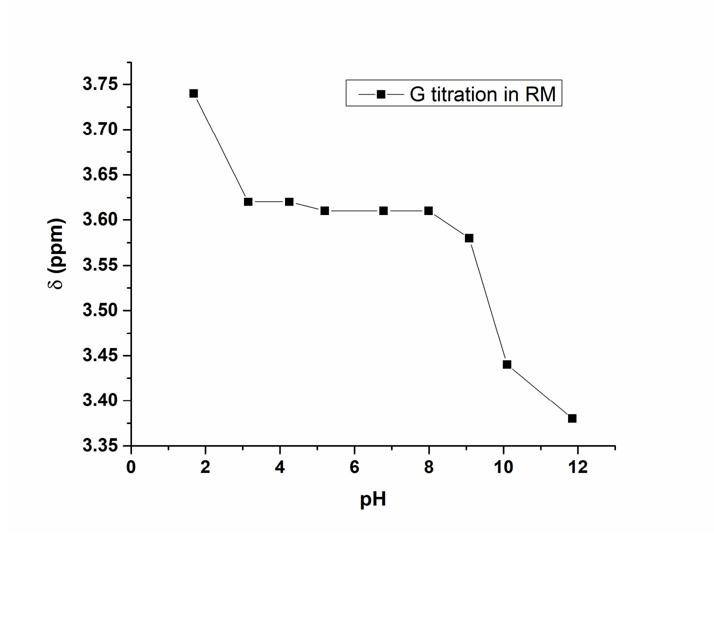

Figure S7. ^{1}H NMR (400 MHz, Bruker) of deuterium oxide solutions of glycine (200 mM) are shown at various pH values added to 750 mM AOT/isooctane in $w_0$30 RM. Spectra were run in triplicate and one representative series is shown.

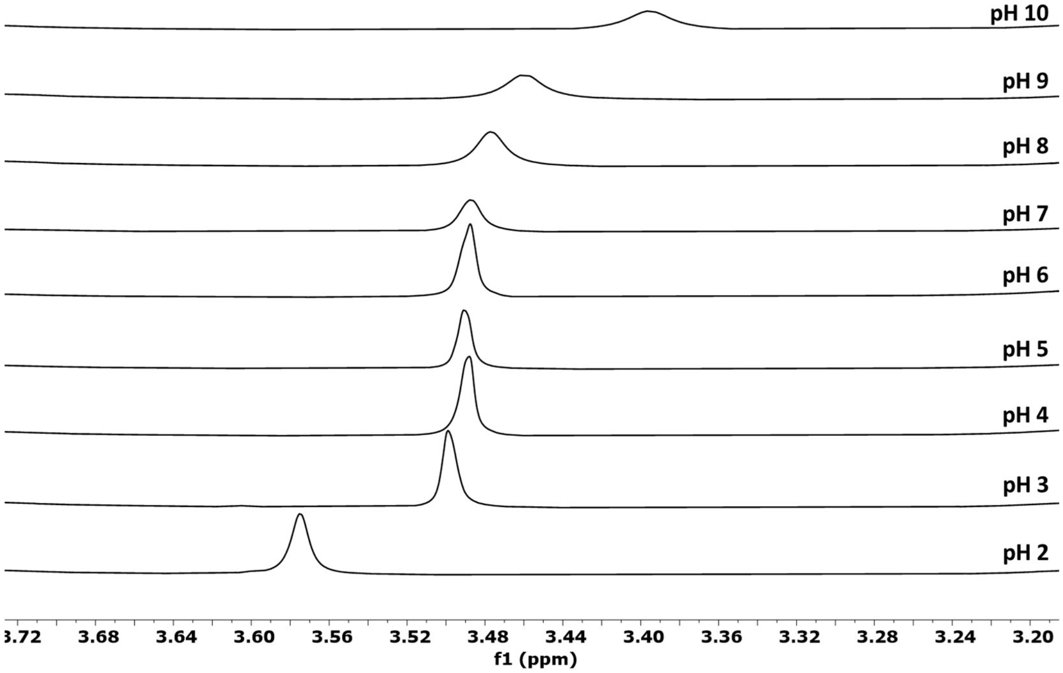

Figure S8. Chemical shifts listed for the ^{1}H NMR spectra at different pH values of glycine (G) in w_0 30 RM (left), a plot of ^{1}H NMR chemical shifts as a function of pH in w_0 30 RM (right). Chemical shifts are obtained from the data shown in Figure S4.

pH	Chemical shift (δ) assignment
2.02	3.574±0.002
3.04	3.5±0.004
4.01	3.486±0.000
5	3.489±0.003
6.03	3.486±0.001
7.1	3.486±0.000
8.06	3.476±0.001
9.01	3.46±0.003
10.07	3.37±0.002

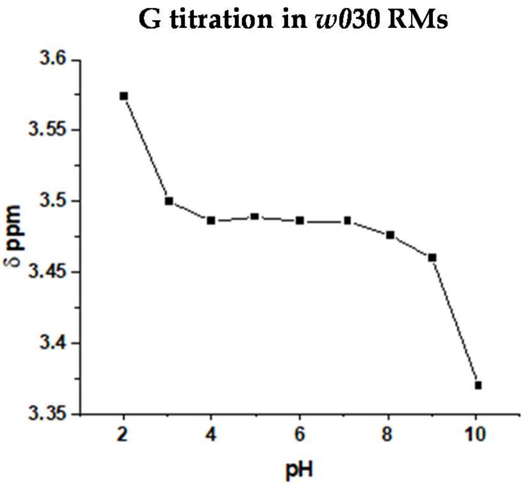

Figure S9. ^{1}H NMR (400 MHz, Bruker) of deuterium oxide solutions of diglycine (200 mM) are shown at various pH values. Samples are referenced externally against DSS. Spectra were run in triplicate and one representative series is shown.

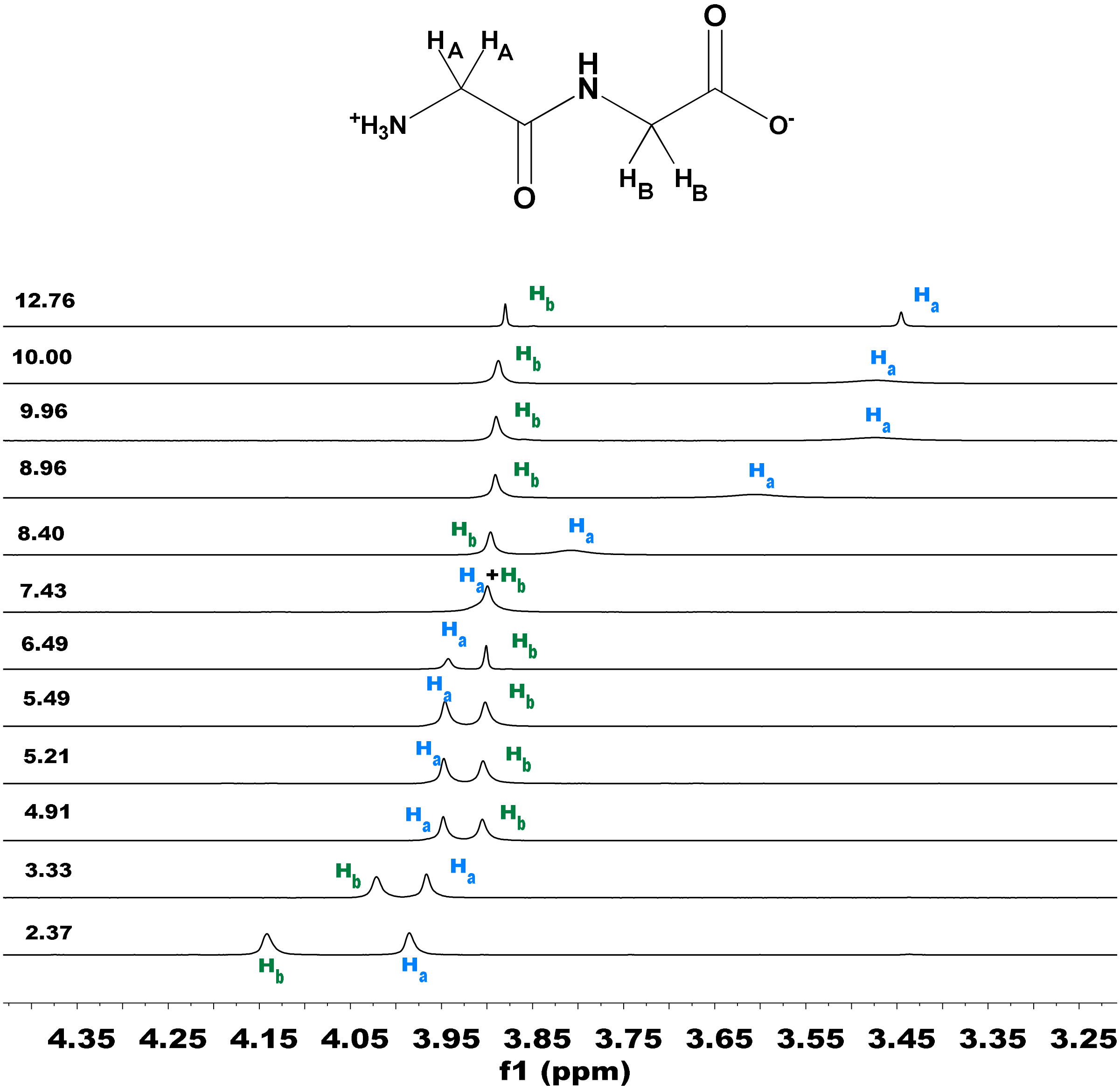

Figure S10. Chemical shifts listed for the ^{1}H NMR spectra at different pH values of diglycine (GG) in D_2O (left), a plot of ^{1}H NMR chemical shifts as a function of pH (right). Chemical shifts are obtained from the data shown in Figure S6.

pH	Chemical shift (δ) assignment	
	Ha	Hb
2.37	4.05±0.003	3.99 ±0.002
3.33	3.93±0.002	3.87±0.002
4.91	3.85±0.001	3.81±0.004
5.21	3.85±0.002	3.81±0.003
5.49	3.85±0.001	3.81±0.003
6.49	3.85±0.004	3.81±0.002
7.43	3.81±0.003	3.81±0.000
8.40	3.71±0.002	3.80±0.002
8.96	3.51±0.005	3.79±0.000
9.96	3.37±0.003	3.79±0.000
10.00	3.37±0.001	3.79±0.000
12.76	3.35±0.004	3.79±0.000

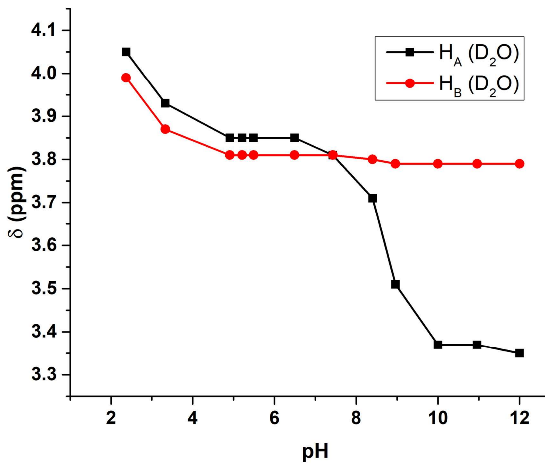

Figure S11. ^{1}H NMR (400 MHz, Bruker) of deuterium oxide solutions of glycine (200 mM) are shown at various pH values added to 750 mM AOT/isooctane in $w_0$10 RMs. Samples are referenced against isooctane at 0.904 ppm. Spectra were run in triplicate and one representative series is shown.

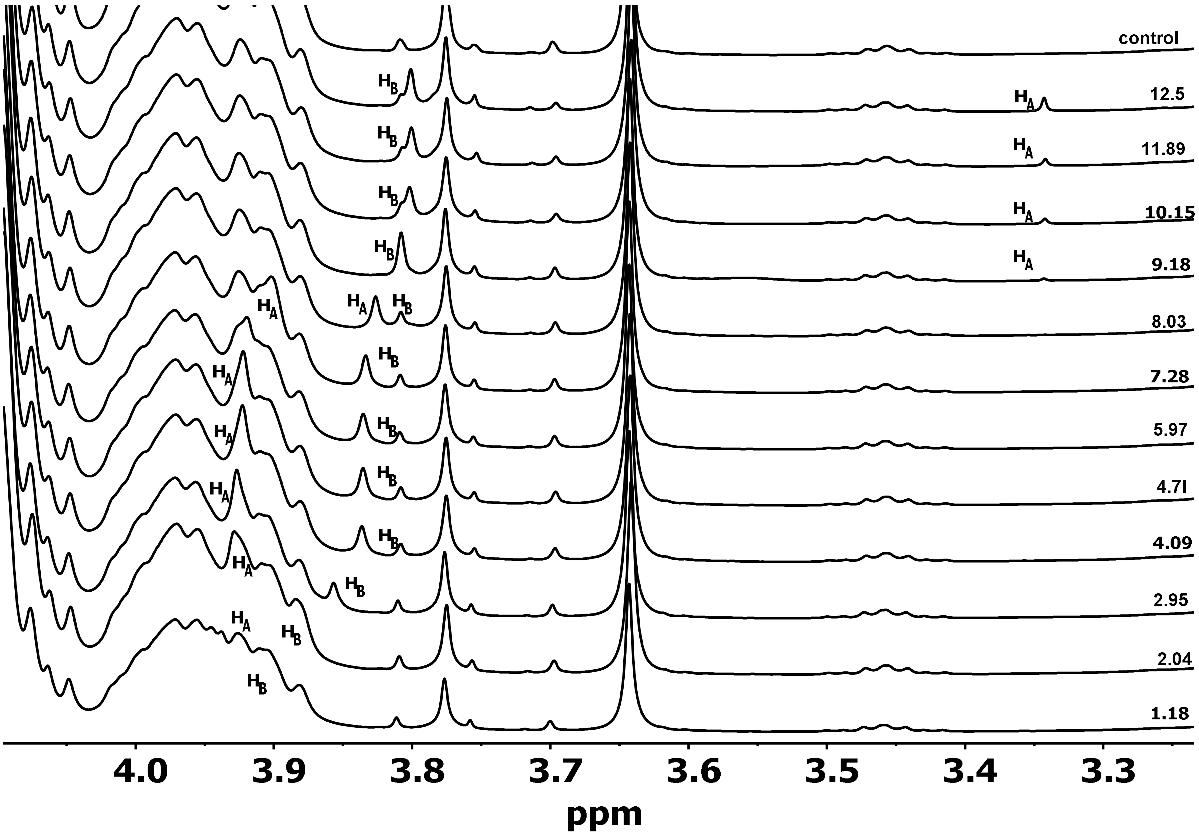

Figure S12. Chemical shifts listed for the ¹H NMR spectra at different pH values of diglycine (GG) in *w₀*10 RM (left), a plot of ¹H NMR chemical shifts as a function of pH in RM (right). Chemical shifts are obtained from the data shown in Figure S8.

pH	Chemical shift (δ) assignment	
	Ha	Hb
1.18	3.93±0.000	3.91±0.001
2.04	3.93±0.000	3.88±0.001
2.95	3.93±0.000	3.86±0.001
4.09	3.92±0.001	3.84±0.000
4.7	3.92±0.000	3.84±0.000
5.97	3.92±0.001	3.84±0.000
7.28	3.90±0.001	3.83±0.001
8.03	3.83±0.002	3.83±0.000
9.18	3.35±0.001	3.81±0.000
10.15	3.34±0.000	3.80±0.001
11.89	3.34±0.000	3.80±0.000
12.5	3.34±0.000	3.80±0.000

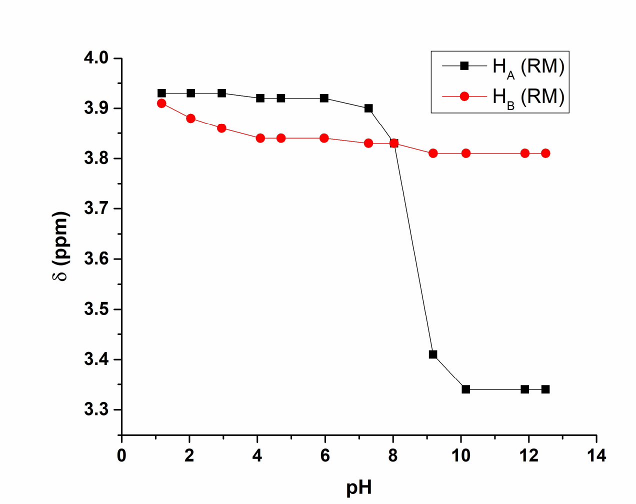

Figure S13. ^{1}H NMR (400 MHz, Bruker) of deuterium oxide solutions of triglycine (200 mM) are shown at various pH values. Samples are referenced externally against DSS. Spectra were run in triplicate and one representative series is shown.

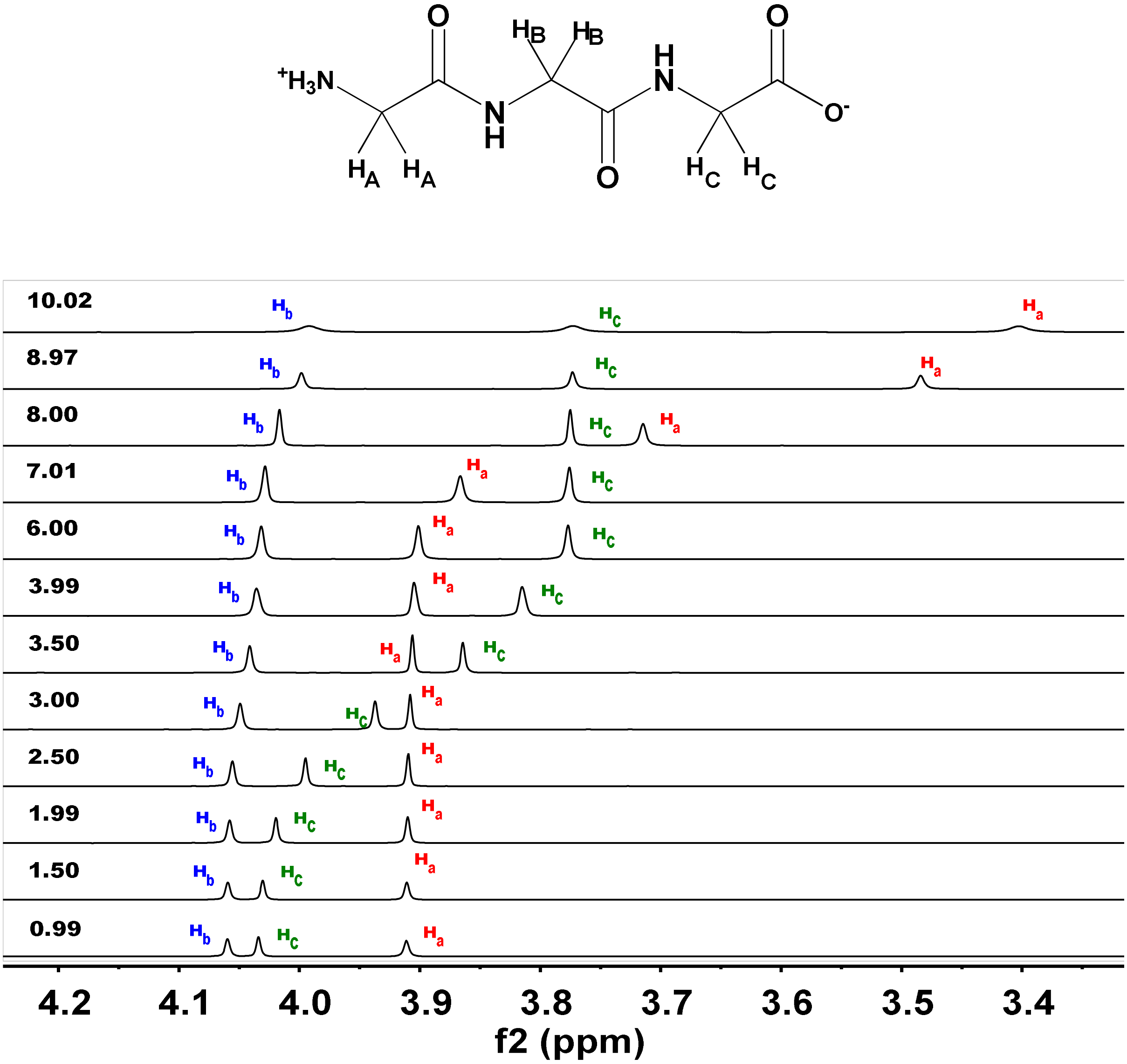

Figure S14. Chemical shifts listed for the ^{1}H NMR at different pH values of triglycine (GGG) (left), a plot of ^{1}H NMR chemical shifts as a function of pH (right). Chemical shifts are obtained from the data shown in Figure S10.

pH	Chemical shift (δ) assignment		
	Ha	Hb	Hc
0.99	3.91±0.000	4.03±0.001	4.06±0.000
1.50	3.91±0.000	4.03±0.001	4.06±0.000
1.99	3.91±0.000	4.02±0.001	4.06±0.000
2.50	3.91±0.000	3.99±0.001	4.06±0.000
3.00	3.91±0.000	3.94±0.001	4.05±0.001
3.50	3.91±0.000	3.86±0.002	4.04±0.001
3.99	3.90±0.001	3.82±0.002	4.04±0.000
6.00	3.90±0.000	3.78±0.000	4.03±0.001
7.01	3.87±0.001	3.78±0.001	4.03±0.001
8.00	3.71±0.002	3.78±0.001	4.02±0.000
8.97	3.48±0.003	3.77±0.001	4.00±0.000
10.02	3.40±0.002	3.77±0.001	3.99±0.001

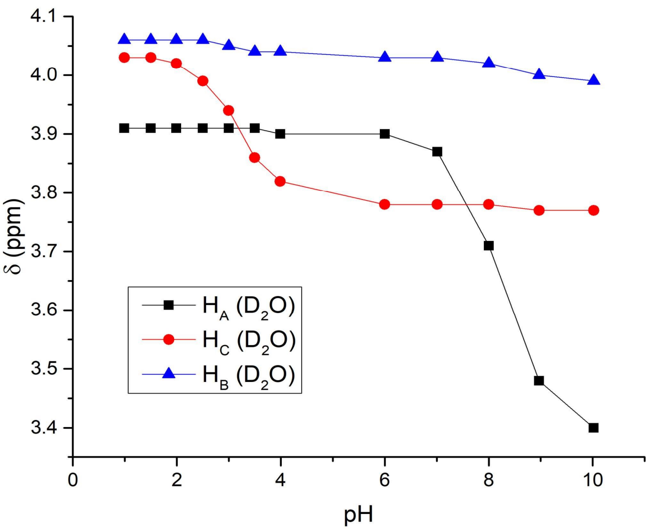

Figure S15. Representative subtraction of the ^{1}H NMR spectra of GGG in w_0 10 RM. ^{1}H NMR (400 MHz, Bruker) of deuterium oxide solutions of triglycine (200 mM) are shown at various pH values added to 750 mM AOT/isooctane in w_0 10 RMs. Samples are referenced against isooctane at 0.904 ppm. Spectra were run in triplicate and one representative series is shown. Subtraction was carried out as described in the experimental section.

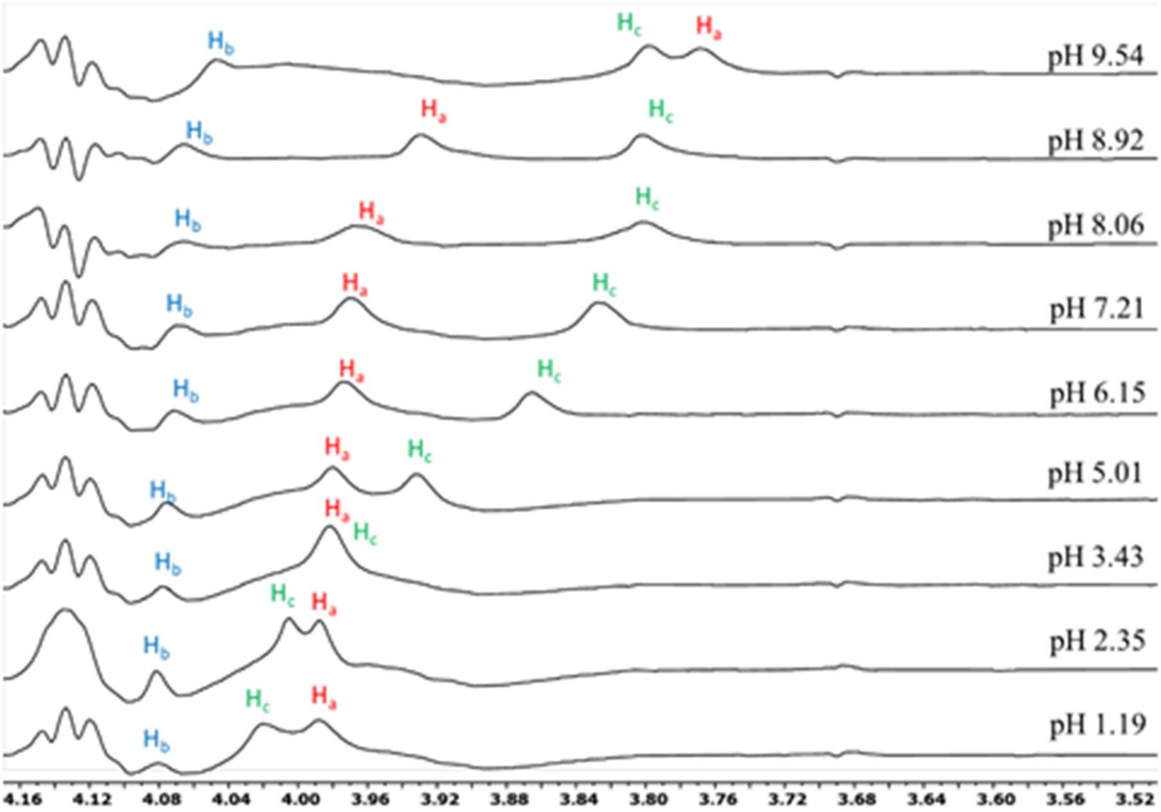

Figure S16. Chemical shifts listed for the ^{1}H NMR at different pH values of triglycine (GGG) (left), a plot of ^{1}H NMR chemical shifts as a function of pH (right). Chemical shifts are obtained from the data shown in **Fig. S14**.

pH	Chemical shift (δ) assignment		
	Ha	Hb	Hc
1.50	3.99±0.001	4.09±0.000	4.03±0.001
1.99	4.00±0.000	4.09±0.000	4.02±0.002
2.50	4.00±0.000	4.09±0.000	4.00±0.002
3.00	4.00±0.001	4.09±0.000	3.95±0.002
3.50	3.98±0.001	4.09±0.000	3.86±0.003
3.99	3.97±0.002	4.09±0.002	3.83±0.003
6.00	3.95±0.002	4.06±0.001	3.79±0.002
7.01	3.92±0.002	4.06±0.001	3.78±0.002
8.00	3.77±0.003	4.06±0.000	3.77±0.001
8.97	3.75±0.001	4.06±0.000	3.77±0.000
10.02	3.74±0.001	4.05±0.002	3.77±0.000

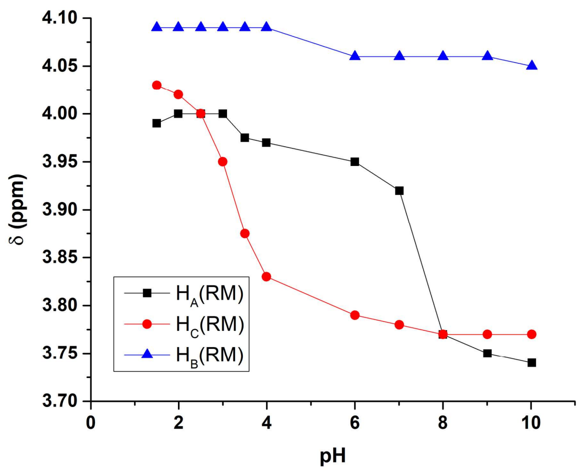

Figure S17. ^{1}H NMR (400 MHz, Bruker) of deuterium oxide solutions of tetraglycine (200 mM) are shown at various pH values. The pH values are measured directly in D_2O,and are not a reflection of the solution pD (pD = pH + 0.4) {Crans, 2011 #168; Koehn, 2017 #169; Samart, 2014 #170}. pH values were adjusted with DCl (1M) and NaOD (1M). Samples are referenced externally against DSS. Spectra were run in triplicate and one representative series is shown.

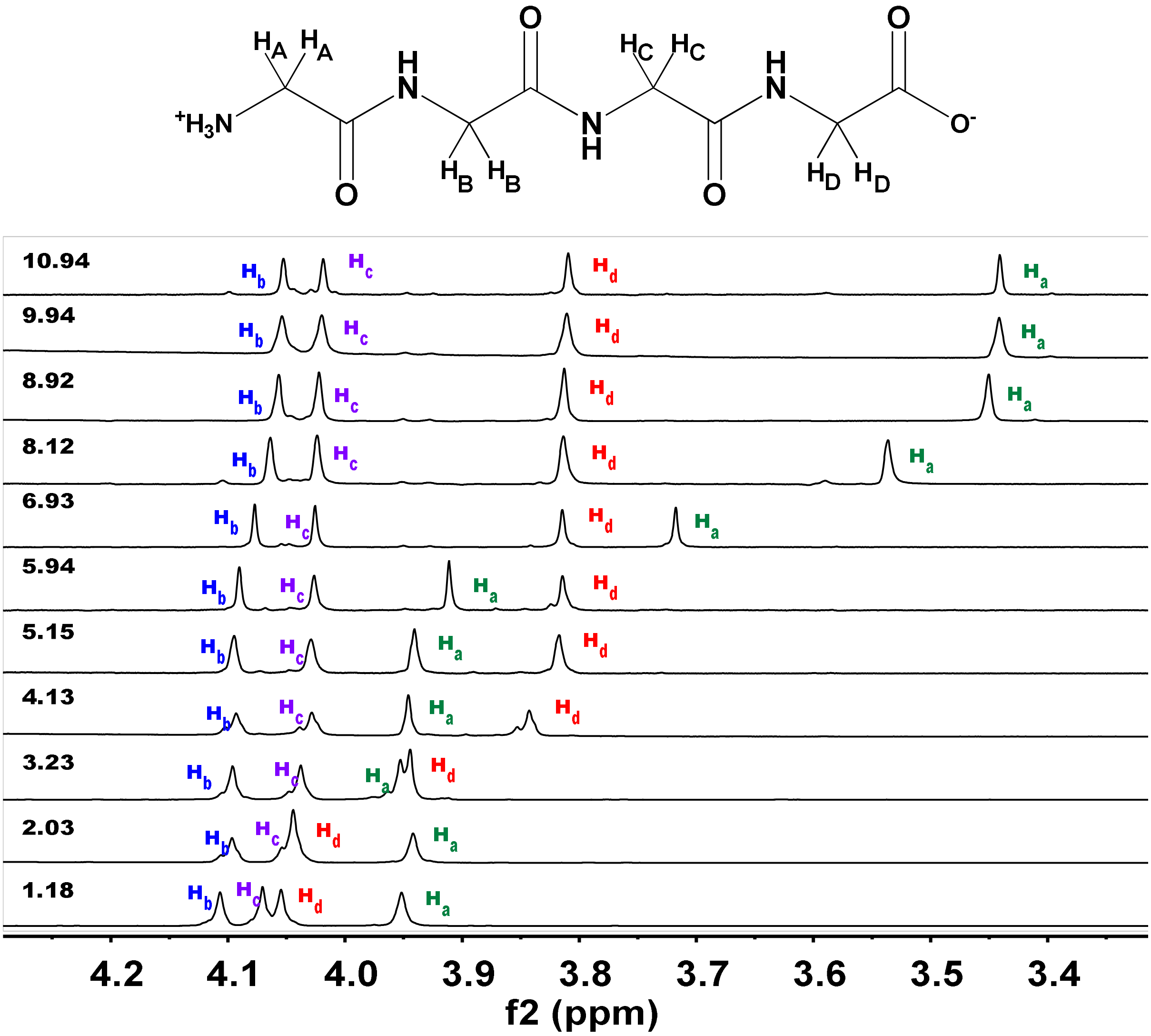

Figure S18. Chemical shifts listed for the ^{1}H NMR at different pH values of tetraglycine (GGGG) (left), a plot of ^{1}H NMR chemical shifts as a function of pH (right). Chemical shifts are obtained from the data shown in **Fig. S16.**

pH	Chemical shift (δ) assignment			
	Ha	Hb	Hc	Hd
1.18	4.11±0.000	4.07±0.002	4.05±0.001	3.95±0.002
2.03	4.10±0.001	4.04±0.002	4.04±0.002	3.94±0.000
3.23	4.10±0.001	3.95±0.001	4.04±0.003	3.94±0.000
4.13	4.09±0.001	3.85±0.001	4.03±0.001	3.94±0.000
5.15	4.09±0.002	3.82±0.001	4.03±0.001	3.94±0.003
5.94	4.09±0.002	3.81±0.000	4.03±0.001	3.91±0.002
6.93	4.08±0.002	3.81±0.000	4.03±0.001	3.72±0.002
8.12	4.06±0.001	3.81±0.000	4.02±0.001	3.54±0.004
8.92	4.06±0.001	3.81±0.000	4.02±0.001	3.45±0.002
9.94	4.05±0.001	3.81±0.000	4.02±0.001	3.44±0.001
10.94	4.05±0.002	3.81±0.000	4.02±0.001	3.44±0.000

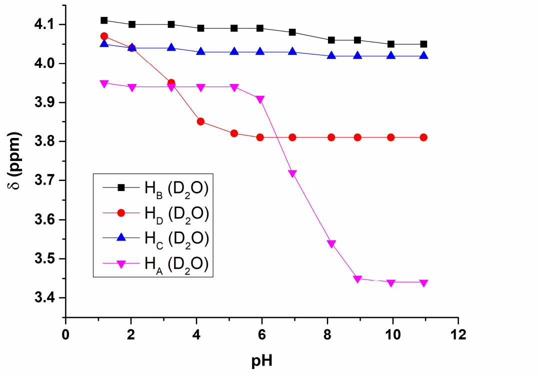

Figure S19. Representative spectrum showing subtraction technique utilized to elucidate GGGG peaks overlapped by AOT peaks at pH 7. Samples are referenced against isooctane at 0.904 ppm. Spectra were run in triplicate and one representative series is shown. Subtraction was carried out as described in the experimental section.

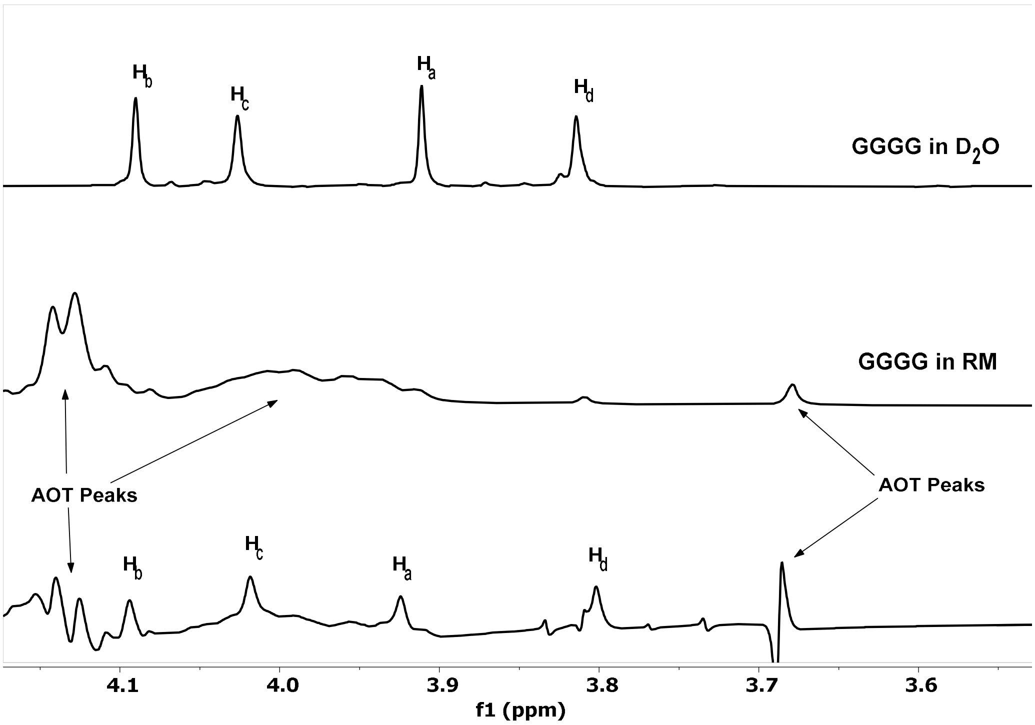

Figure S20. Chemical shifts listed for the ^{1}H NMR spectra at different pH values of tetraglycine (GGGG) (left), a plot of ^{1}H NMR chemical shifts as a function of pH (right). Chemical shifts are obtained from the data shown in **Fig. S18.**

pH	Chemical shift (δ) assignment			
	Ha	Hb	Hc	Hd
0.99	3.99± 0.000	4.10± 0.000	4.03± 0.000	4.04± 0.002
2.03	3.99± 0.000	4.10± 0.000	4.03± 0.000	3.98± 0.003
2.98	3.99± 0.001	4.10± 0.000	4.03± 0.001	3.92± 0.003
3.98	3.98± 0.001	4.10± 0.000	4.02± 0.002	3.82± 0.003
4.8	3.98± 0.001	4.10± 0.000	4.02± 0.002	3.8±0 .002
6.04	3.97± 0.003	4.10± 0.002	4.02± 0.002	3.8±0 .002
7.04	3.93± 0.002	4.09± 0.002	4.02± 0.002	3.8±0 .000
8.01	3.74± 0.004	4.08± 0.003	4.01± 0.002	3.8±0 .000
8.94	3.57± 0.004	4.06± 0.002	4.01± 0.000	3.8±0 .000
10.04	3.54± 0.002	4.05± 0.001	4.01± 0.000	3.8±0 .000

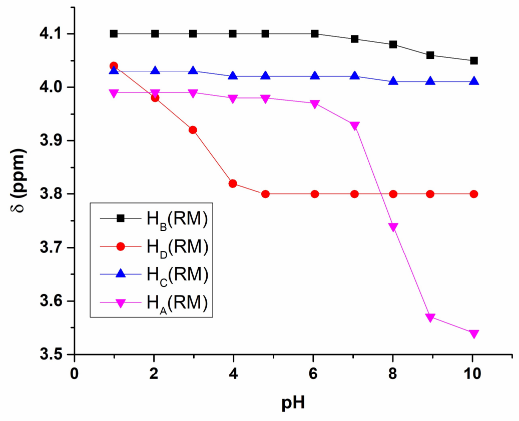

Table S1. Percent difference between the area per molecule of DPPC control monolayers and monolayers with glycine in the subphase. Values were taken at every 5 mN/m of surface pressure from the average compression isotherm of at least three trials. The shaded columns at 30 and 35 mN/m represent what is commonly thought of as physiological surface pressure.

	5 mN/m	10 mN/m	15 mN/m	20 mN/m	25 mN/m	30 mN/m	35 mN/m	40 mN/m	45 mN/m	50 mN/m	55 mN/m
pH 4.0	11.3 ± 7.0	11.3 ± 5.8	8.2 ± 4.6	4.4 ± 3.5	3.1 ± 3.8	0.7 ± 3.8	-1.0 ± 4.0	-2.1 ± 5.0	-2.7 ± 5.4	-3.2 ± 6.5	-4.4 ± 10.1
pH 6.0	14.3 ± 20.2	23 ± 21.0	16.1 ± 12.0	12.3 ± 9.1	8.6 ± 5.4	7.3 ± 5.0	6.7 ± 5.2	6.2 ± 5.5	5.4 ± 6.0	4.6 ± 6.3	1.6 ± 6.1
pH 7.0	18.0 ± 2.9	24.0 ± 5.4	16.2 ± 4.5	10.6 ± 2.1	7.8 ± 1.1	5.2 ± 0.9	3.8 ± 2.6	2.7 ± 3.4	2.0 ± 4.1	2.3 ± 5.3	2.0 ± 8.0
pH 8.0	7.7 ± 18.0	10.3 ± 8.0	5.3 ± 4.6	2.4 ± 5.7	-0.3 ± 2.2	-0.9 ± 1.7	-1.6 ± 2.9	-2.0 ± 3.6	-2.5 ± 4.0	-2.9 ± 4.1	-3.6 ± 4.3
pH 9.0	-0.8 ± 8.1	-2.5 ± 4.8	-2.7 ± 4.1	-2.3 ± 3.7	-2.3 ± 3.3	-2.8 ± 3.0	-2.7 ± 2.7	-2.0 ± 2.0	-1.9 ± 1.9	-1.8 ± 1.9	-2.2 ± 2.1

Table S2. Percent difference values and errors between DPPE control monolayers and DPPE monolayers where glycine was present in the subphase. Values were taken every 5 mN/m of surface pressure from the average compression isotherm of at least three trials. The shaded columns of 30 and 35 mN/m represent what is commonly thought of us physiological surface pressure.

	5 mN/m	10 mN/m	15 mN/m	20 mN/m	25 mN/m	30 mN/m	35 mN/m	40 mN/m	45 mN/m	50 mN/m
pH 4.0	18.6 ± 6.5	14.6 ± 7.2	12.1 ± 6.0	10.5 ± 5.7	7.6 ± 4.8	5.3 ± 3.5	3.2 ± 3.0	2.6 ± 2.9	2.5 ± 3.4	1.7 ± 3.8
pH 6.0	16.7 ± 3.7	13.9 ± 3.8	11.4 ± 4.1	9.1 ± 3.2	8.2 ± 3.0	6.6 ± 3.8	5.5 ± 4.3	5.0 ± 5.2	4.1 ± 6.6	4.0 ± 6.1
pH 7.0	4.9 ± 5.6	1.5 ± 5.1	-1.4 ± 1.7	-2.2 ± 2.3	-2.0 ± 3.4	-2.3 ± 3.6	-2.8 ± 3.2	-3.1 ± 2.8	-3.4 ± 3.8	-2.3 ± 9.9
pH 8.0	17.8 ± 6.3	13.8 ± 5.4	10.9 ± 4.6	8.5 ± 3.3	6.4 ± 2.1	5.2 ± 1.4	4.3 ± 1.3	3.7 ± 1.7	3.4 ± 2.0	2.4 ± 3.0
pH 9.0	1.8 ± 1.4	1.9 ± 1.3	2.1 ± 1.2	2.3 ± 1.1	2.3 ± 1.1	2.3 ± 1.1	2.4 ± 1.2	2.4 ± 1.3	2.4 ± 1.3	1.8 ± 1.6
